# Role of DegQ in differential stability of flagellin subunits in *Vibrio vulnificus*

**DOI:** 10.1038/s41522-021-00206-7

**Published:** 2021-04-08

**Authors:** You-Chul Jung, Mi-Ae Lee, Han-Shin Kim, Kyu-Ho Lee

**Affiliations:** 1 Department of Life Science, Sogang University, Seoul, South Korea; 2 Department of Environmental Science, Hankuk University of Foreign Studies, Yongin, South Korea; 3 Present Address: Graduate School of Biotechnology, Kyung Hee University, Yongin, Gyeonggi-Do South Korea; 4 Present Address: Korean Peninsula Infrastructure Special Committee, Korea Institute of Civil Engineering and Building Technology, Goyang, Gyeonggi-Do South Korea

**Keywords:** Biofilms, Bacteriology

## Abstract

Biofilm formation of *Vibrio vulnificus* is initiated by adherence of flagellated cells to surfaces, and then flagellum-driven motility is not necessary during biofilm maturation. Once matured biofilms are constructed, cells become flagellated and swim to disperse from biofilms. As a consequence, timely regulations of the flagellar components’ expression are crucial to complete a biofilm life-cycle. In this study, we demonstrated that flagellins’ production is regulated in a biofilm stage-specific manner, via activities of a protease DegQ and a chaperone FlaJ. Among four flagellin subunits for *V. vulnificus* filament, FlaC had the highest affinities to hook-associated proteins, and is critical for maturating flagellum, showed the least susceptibility to DegQ due to the presence of methionine residues in its DegQ-sensitive domains, ND1 and CD0. Therefore, differential regulation by DegQ and FlaJ controls the cytoplasmic stability of flagellins, which further determines the motility-dependent, stage-specific development of biofilms.

## Introduction

An opportunistic foodborne pathogen, *Vibrio vulnificus*, causes high risk of fatal septicemia or gastroenteritis in humans with immunocompromised states, chronic liver disease, hemochromatosis, or diabetes^1^. Various factors have been experimentally proven to play roles in eliciting its virulence to hosts. In addition, the abilities to perform specific activities endowing this pathogen with metabolic and behavioral advantages when interacting with hosts are also considered as one of the pathogen’s virulence factors^2–4^. One of such abilities is the capacity to efficiently form biofilms, which has been found to be important in the pathogenicity of various bacteria including *V. vulnificus*^5,6^. Biofilm formation is initiated by bacterial adherence on given surfaces and continues to develop into mature structures with appropriate sizes, which are dependent upon the species of bacteria and the environmental parameters in incubation conditions. The developmental process for biofilm formation is then followed by bacterial cells departing from biofilm structures to the planktonic phase^7^. The initial adherence and final dispersal stages of biofilm formation are highly facilitated if bacteria are able to swim by virtue of cellular motility mediated by bacterial flagellum^8,9^. In contrast, swimming motility is not required for maturation of biofilms, and thus most bacterial cells during the maturation stage are non-flagellated^10^. Therefore, flagellar expression and assembly should be dynamically and tightly regulated in response to biofilm stages during the entire period of the biofilm-forming process.

The bacterial flagellum, a complex self-assembling appendage, is composed of the basal body, the hook, and the filament^11^. The filament is composed of up to 20,000 subunits of a single flagellin or multiple kinds of flagellins. In case of *V. vulnificus*, multiple flagellin subunits, FlaA (39.9 kDa), FlaB/D (40.0 kDa), and FlaC (41.1 kDa), produced from four genes constitute its filament^12^. A growing tip of filament is capped with the scaffold protein, FliD, in which the secreted flagellins are permitted to be folded and polymerized^13^. The other tip of filament is connected with the hook-associated proteins (HAPs), FlgK and FlgL, which are structural adaptors located between the filament and hook^14^. The hook is a joint to connect the filament with the basal body consisting of a rotor and stator, which are embedded in the cytoplasmic membrane^15^.

The formation of the flagellar structure is under strict spatio-temporal regimes controlled by sequential and hierarchical transcription of genes in the flagellar gene clusters. Moreover, the flagellar assembly is also guided by various chaperone proteins^11^. One of the well-known chaperones for flagellar biogenesis is FlaJ (or FliS), which functions to export nascent flagellins in cytoplasm to the secretion channel in the basal body^16^. FlaJ binds the C-terminal region of flagellin to form a flagellin-FlaJ heterodimer^17^, which subsequently interacts with FlhA in the flagellum-specific secretion channel^18^. In addition to FlaJ, other flagellar chaperones, such as FlgN and FliT, have been proposed to act as substrate-specific export chaperones, which facilitate incorporation of FlgK/FlgL and FliD to the tips of a filament, respectively, in *Salmonella* Typhimurium^19^. In *Campylobacter jejuni*, a flagellar stator-specific chaperone has been identified to be FlgX, which ensures the integrity of the MotAB stator unit against proteolysis by the inner membrane protease, FtsH^20^.

**Fig. 1:**
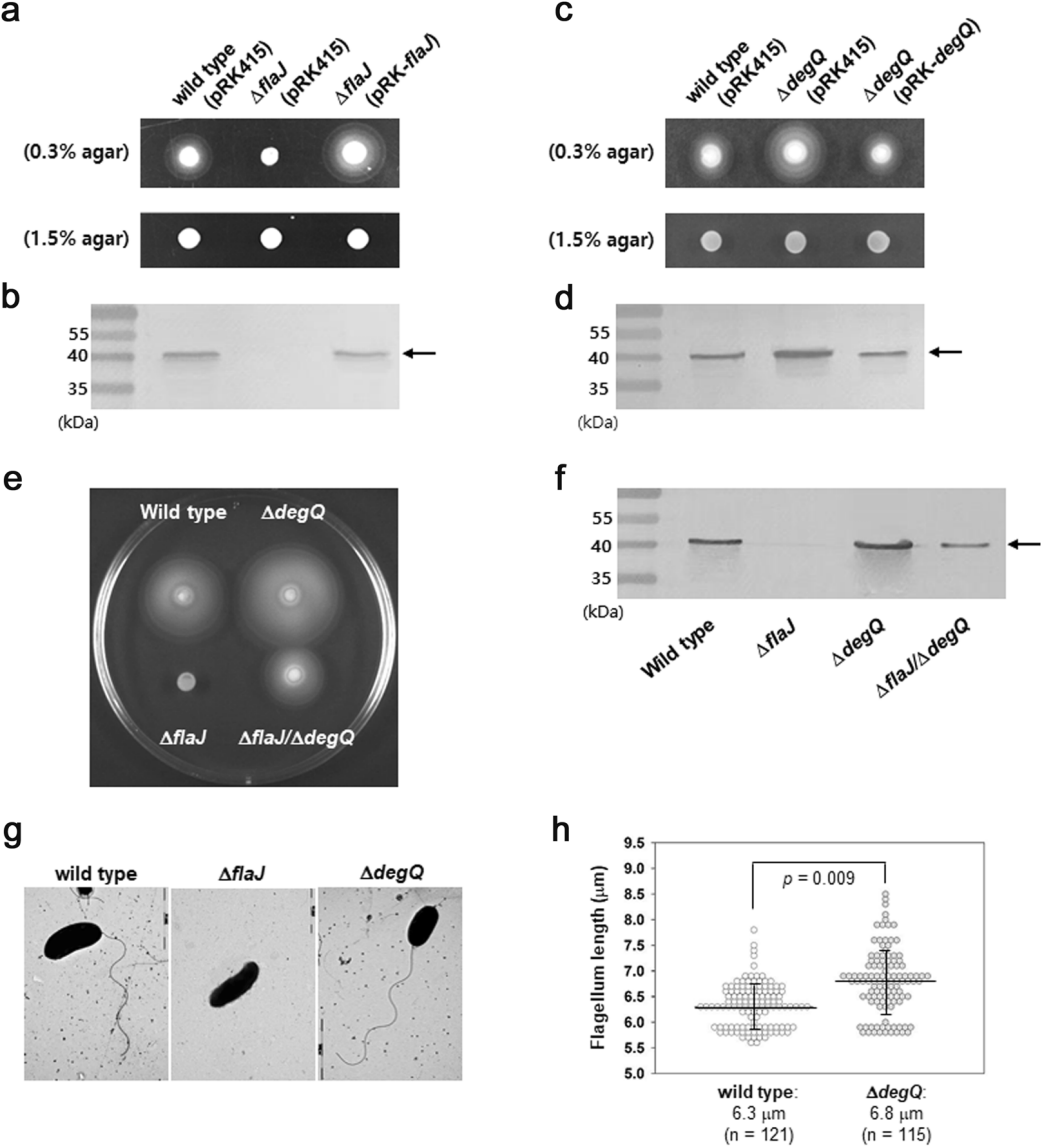
Effects of the mutations in *flaJ* or *degQ* genes on flagellins, flagella, and motility. **a** Effect of FlaJ on swimming motility on the soft-agar plate. Five microliters of the freshly grown cultures (at an OD of 1.0) of *V. vulnificus* strains of wild type and Δ*flaJ* carrying a broad-host-range vector (pRK415) or a pRK415 harboring the intact *flaJ* gene (pRK-*flaJ*) were spot-inoculated on a soft-agar plate made of LBS containing 0.3% agar, tetracycline (3 μg/ml), and IPTG (1.0 mM), and incubated at 30 °C for 6 h. As a control for the inoculum size of each strain, the same volumes of the bacterial suspensions were spotted on the LBS-1.5% agar plate supplemented with tetracycline (3 μg/ml) and IPTG (1.0 mM). **b** Effect of *flaJ* deletion on the cellular levels of flagellins. Cell lysates were prepared from the wild type carrying pRK415, Δ*flaJ* carrying a pRK415, and Δ*flaJ* carrying a pRK415- *flaJ*. Ten micrograms of the crude cell extracts were subjected to SDS-PAGE and subsequently western blot analysis using the polyclonal antibodies reacting with the flagellins produced by *V. vulnificus*, as previously described^12^. An arrow indicated the *V. vulnificus* flagellin subunits. **c** Effect of DegQ on swimming motility on the soft-agar plate. *V. vulnificus* strains of wild type carrying pRK415 and Δ*degQ* carrying pRK415 or pRK-*degQ* were spot-inoculated on the LBS plates containing agar at a concentration of 0.3 or 1.5%, as described in (**a**). **d** Effect of *degQ* deletion on the cellular levels of flagellins. Ten micrograms of cell extracts of wild type carrying pRK415, Δ*degQ* carrying pRK415, and Δ*degQ* carrying pRK415-*degQ* were subjected to SDS-PAGE and western blot analysis, as described in (**b**). **e**, **f** Effects of *flaJ*/*degQ*-double mutations on motility and flagellin levels. *V. vulnificus* strains of wild type, Δ*flaJ*, Δ*degQ*, and Δ*flaJ*/Δ*degQ* were spot-inoculated on a soft-agar plate and incubated at 30 °C for 8.5 h (**e**), as described in (**a**). Ten micrograms of cell extracts of the four strains were subjected to SDS-PAGE and western blot analysis (**f**), as described in (**b**). **g**, **h** Effects of FlaJ and DegQ on flagellation. Individual cells of *V. vulnificus* strains of wild type, Δ*flaJ*, and Δ*degQ* were observed under TEM to examine the polar flagellum (**g**), as described in “Methods”. Lengths of flagella on the TEM images were measured from 121 cells of wild type and 115 cells of Δ*degQ* (**h**). The distribution of flagellar lengths was plotted with the medians (marked with a horizontal line) and their standard deviations (marked with a vertical line) of each strain and the *P*-value derived from comparison of two strains (Student’s *t*-test).

From a preliminary investigation of the motilities of various mutant strains of *V. vulnificus*, a Δ*flaJ* mutant was found to be non-motile in soft-agar plate and contained a non-detectable level of the cytoplasmic flagellin (see Fig. 1). These findings led us to speculate that nascent flagellin polypeptides in cytoplasm were under intense proteolytic pressure if a flagellin-specific chaperone FlaJ was not present in the cytoplasm. Therefore, so-far unidentified protease(s), which is able to specifically proteolyze flagellins, might be a critical regulator counteracting FlaJ activity at the level of post-translation. In the present study, we identified the protease involving the flagellin proteolysis and presented its differential proteolytic characteristics towards four kinds of flagellin subunits found in *V. vulnificus*^12^. The overall effect of the differential proteolysis of different kinds of flagellins on filament assembly was examined.

## Results

### Effects of deletions of *flaJ* and *degQ* genes on flagellum-mediated phenotypes of *V. vulnificus*

The genomes of *V. vulnificus* have been shown to have an ORF (VVMO6_00813) homologous to the *flaJ* (or *fliS*) encoding the flagellin-specific chaperone protein found in other bacterial species, which facilitates the export of flagellins to extracellular milieu and prevents their polymerization in the cytoplasm^17^. To elucidate the role of FlaJ in *V. vulnificus*, the swimming ability of Δ*flaJ* mutant^12^ was tested on a soft-agar plate. As expected, this mutant lost swimming motility, but recovered the ability if the intact *flaJ* gene was complemented by conjugating pRK-*flaJ* (Fig. 1a). Non-motile phenotype of Δ*flaJ* was due to the absence of a polar flagellum in this mutant (Fig. 1g). Interestingly, any cellular flagellin in Δ*flaJ* was not detected in a western blot of its lysate using the polyclonal antibodies reacting with four flagellins of *V. vulnificus*, i.e., FlaA, FlaB, FlaC, and FlaD^12^. In addition, cellular level of the flagellins in Δ*flaJ* was restored to that in wild type, when complemented with the *flaJ* gene (Fig. 1b). Non-detectable level of flagellin in the mutant defective in FlaJ suggested that the additional role of FlaJ chaperone in cytoplasmic flagellin stability would be played at the level of post-translation.

To identify the factor(s) impairing the protein stability of flagellins, a protease was sought that specifically affected the cellular levels of flagellins and swimming motility through screening of various protease/chaperone mutants of *V. vulnificus* (Supplementary Fig. 1). A mutant deficient in *degQ* gene (VVMO6_02600), which encodes a serine protease belonging to the HtrA family^21^, exhibited increased motility compared to wild type (Fig. 1c). In addition, the cellular level of flagellins in Δ*degQ* was highly increased compared to wild type (Fig. 1d). The increased levels of motility and flagellins shown by the *degQ* mutant were returned to the levels of wild type when the mutant was complemented with the intact *degQ* gene. To examine the cellular localization of DegQ, immunofluorescence assays were performed using the antibodies specific to DegQ, OmpU, or PhoA (Supplementary Fig. 2). When the locations of visualized DegQ were compared with those of an outer membrane protein OmpU^22^, considerable amounts of DegQ were present in the cytoplasm, in which OmpU proteins were not obviously observed. DegQ proteins colocalized with OmpU appeared to be in the periplasm, since a periplasmic protein PhoA^23^ was also colocalized where OmpU proteins were visualized. In addition, when Δ*flaJ* was further mutated at its *degQ* locus to produce a mutant defective in both *flaJ* and *degQ* (Δ*flaJ*/Δ*degQ*), this double mutant showed to have cellular flagellin subunits and gain the swimming ability (Fig. 1e). It demonstrated the importance of DegQ in determining the flagellins’ stability. However, it exhibited less levels of flagellins and motility than those shown by the wild type (Fig. 1e and f), which might suggest the presence of another factor proteolyzing the flagellins in *V. vulnificus*.

Transmission electron microscope (TEM) images of the Δ*degQ* cells revealed that they have slightly longer flagella than the wild-type cells (Fig. 1g and h). Measurement of the flagellar lengths shown in the TEM images of wild type (*n* = 121) and Δ*degQ* (*n* = 115) revealed average lengths of 6.3 and 6.8 μm, respectively (*P* = 0.009, Student’s *t*-test). Increased motility of the Δ*degQ*, shown in Fig. 1c, appeared to be attributable to an earlier achievement of motility ability and more flagellated individuals in a spotted population of Δ*degQ* than the wild type. Observation of the swimming motility of wild type and Δ*degQ* on a soft-agar plate showed that Δ*degQ* began to swim at the early incubation time (e.g., at 1–2 h post-inoculation), compared to the wild type (e.g., at 3 h post-inoculation) (Supplementary Fig. 3). Consequently, DegQ activity appears to play an important role in lowering the cytoplasmic stability of flagellins in *V. vulnificus*.

### Involvement of DegQ activity during the biofilm-forming process

**Fig. 2:**
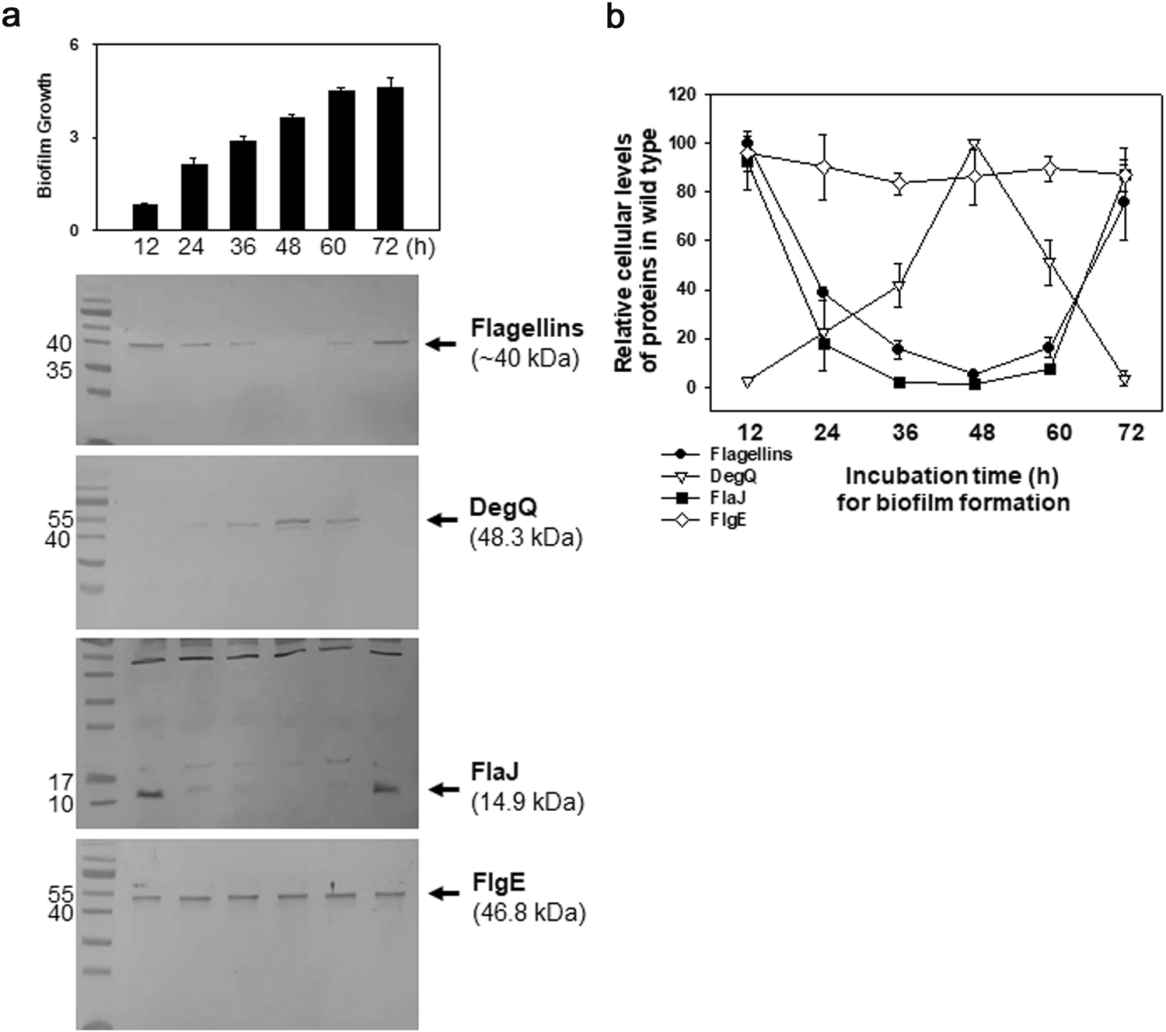
Cellular levels of flagellins, DegQ, and FlaJ during the entire period of biofilm formation. Standard biofilm assays using borosilicate tubes were performed with the wild type carrying pRK415 by inoculating cells in AB-fumarate broth supplemented with tetracycline (3 μg/ml) and incubating at 30 °C for 72 h. Bacterial cells in the biofilm structures were separated from the planktonic cells at the designated incubation time points (i.e., 12, 24, 36, 48, 60, and 72 h). Then, the lysates of biofilm cells were prepared, and 2, 10, 10, and 2 μg of cell extracts were subjected to western blotting analyses using the antibodies specific to flagellins, DegQ, FlaJ, or FlgE, respectively. Resultant protein bands were designated with arrows and their sizes in kDa (**a**). Through densitometric reading of the corresponding bands in wild type, the cellular levels of each protein (relative to the maximal intensity in each blot) were plotted against incubation time points (**b**). The error bars on the graphs represent standard deviation.

To examine the role of DegQ in biofilm formation in a stage-specific manner, the cellular levels of flagellins, DegQ, FlaJ, and FlgE, were monitored in biofilm cells of wild type during the whole biofilm-forming process (Fig. 2a). Western blots showed that the cellular levels of flagellin and FlaJ decreased during 12–48 h, but increased during 60–72 h. In contrast, the cellular levels of DegQ increased during 12–48 h, but decreased during 60–72 h. Cellular levels of the hook FlgE were consistent during all assay periods. The averages of the relative cellular amounts of each protein, quantified by densitometric analysis of the total three independent experiments, were plotted in Fig. 2b. Increased DegQ and decreased FlaJ in the middle of incubation periods for biofilm formation (i.e., 36–60 h) resulted in minimal levels of cytoplasmic flagellins, which were <20% of the flagellins in cells harvested at 12 h. Therefore, the cellular levels of DegQ and FlaJ were differently and independently fluctuating during the periods of biofilm formation, by which the cellular amounts of flagellin subunits were determined by a combination of two opposite activities.

### Role of DegQ in the initial adherence and the final dispersal stages

**Fig. 3:**
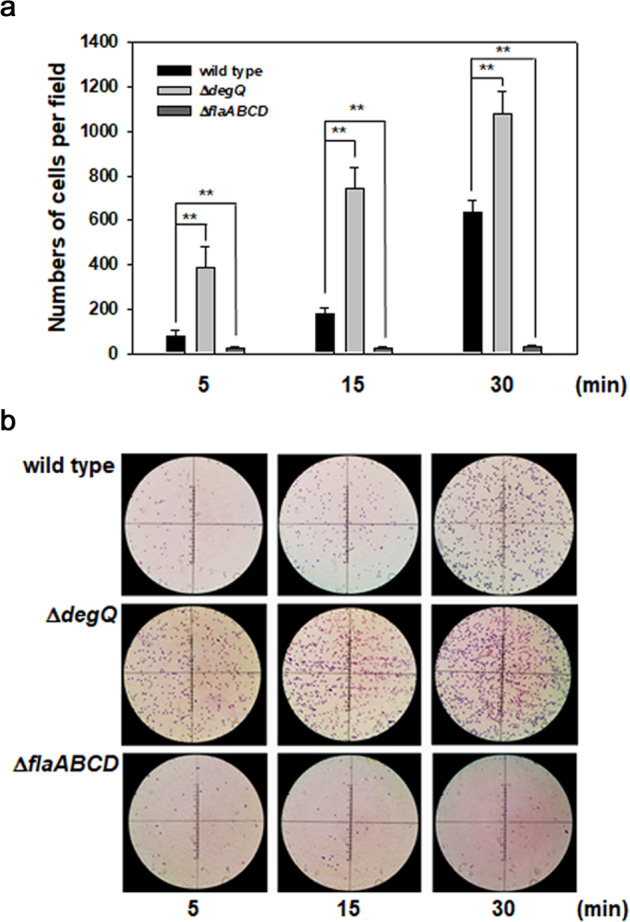
Effect of *degQ* mutation on bacterial adherence on slide glasses. Slide glasses were submerged in flasks containing the AB-fumarate broth inoculated by wild-type or Δ*degQ* strains of *V. vulnificus* at an OD_595_ of 0.2. After 5, 15, and 30 min, the bacterial cells attached on the slide glasses were stained with crystal violet and were counted under the light microscope. To differentiate the bacterial adherence via swimming motility or random contacts to slide glasses, a non-motile mutant of *V. vulnificus*, Δ*flaABCD*, was included as a negative control^12^. Average numbers of adherent cells per a light-microscopic field (at least 18 fields observed) were presented with the error bars representing standard deviations (**a**). The *P-*values for comparison with wild type are indicated (Student’s *t*-test; **P < 0.005). Representative pictures of the slides adhered by three strains for 5, 15, and 30 min were presented (**b**).

To elucidate the role of DegQ in the earlier stage of biofilm formation, Δ*degQ* was inoculated to the AB-fumarate broth submerged with slide glasses to allow bacterial cells to adhere for 30 min. The numbers of cells attached on the slides were counted under a light microscope after the slide glasses collected at 5, 15, and 30 min were stained with crystal violet (Fig. 3). For comparison of its adhering ability on a slide glass, the wild type and an aflagellated mutant, Δ*flaABCD*^12^, were included in the assay. While the numbers of aflagellated, non-motile cells on slides were the basal levels for 30 min, those of the wild type were determined to increase as incubation time increased. The Δ*degQ* cells showed significantly enhanced ability to adhere on the slides, which was approximately 4.9-, 4.2-, and 1.7-times more than the wild-type cells at 5, 15, and 30 min, respectively.

**Fig. 4:**
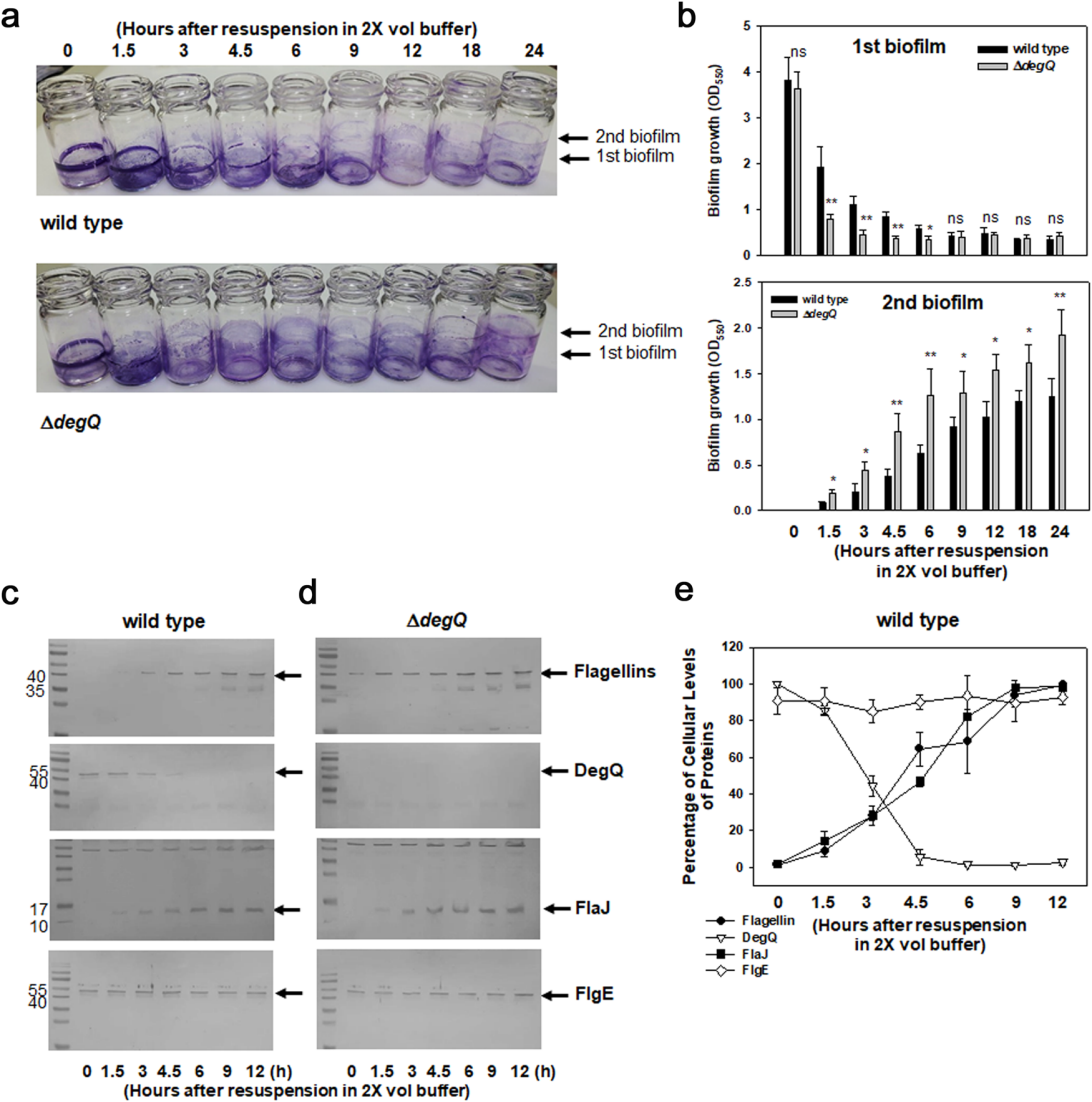
Effect of *degQ* mutation on the cellular levels of flagellins during dispersal from biofilms. **a**, **b** Dispersal of bacterial cells from biofilms. Biofilms formed by the wild-type (upper image) and Δ*degQ* (lower image) in 2 ml AB-fumarate broth for 48 h were further incubated in 4 ml PBS, to allow the second biofilms to be formed around the air–liquid interfaces by the cells dispersed from the first biofilms, as previously described^60^. At various time points during 24 h incubation (0, 1.5, 3, 4.5, 6, 9, 12, 18, and 24 h), the remaining first biofilm and newly formed second biofilm were stained with crystal violet, and the dyes associated with each biofilm were estimated by spectrophotometry at 550 nm (**b**). The error bars on the bar charts represent standard deviation. The *P-*values for the comparison with wild type are indicated (Student’s *t*-test; ns, not significant; *0.005 < *P* < 0.05; **P < 0.005). **c**–**e** Cellular levels of various proteins during the dispersal period. Crude extracts of cells in the first biofilms, which remained in the original positions for the initial 12 h period of the dispersal stage, were subjected to western blotting analyses to monitor the cellular levels of flagellins, DegQ, FlaJ, and FlgE in the wild type (**c**) and Δ*degQ* (**d**). The intensities of resultant protein bands in the wild-type blots were estimated by densitometric reading, and the relative levels of each protein were plotted against incubation time points (**e**). The error bars represent standard deviation.

To differentiate the role of DegQ in the dispersal stage of biofilm formation, the biofilms formed by wild type and Δ*degQ* for 48 h (1st biofilms; Fig. 4a) were resuspended in 2× volume of fresh phosphate-buffered saline (PBS) to allow bacterial cells in the first biofilms to be dispersed and then adhere on the new surfaces at liquid–air interface given by 2× volume of PBS (2nd biofilms; Fig. 4a). Incubation at 30 °C for another 24 h revealed that the first biofilms of the Δ*degQ* mutant became more rapidly diminished than those of the wild type. In contrast, the second biofilms of the mutant were more progressively constructed than those of the wild type (Fig. 4b). The stains associated with the second biofilms formed by the Δ*degQ* mutant were 1.4–2.4-times more than those formed by the wild type.

To examine the cellular levels of DegQ and flagellar components under the condition inducing the dispersal of biofilms, the cells composed of the first biofilms were harvested at the designated time points after the dispersion was allowed (i.e., at 0, 1.5, 3, 4.5, 6, 9, and 12 h post-dispersion), and their cell lysates were prepared for western blot analyses. The western blots using the lysates of wild-type first biofilms demonstrated that the cellular levels of flagellins and FlaJ began to appear as those of DegQ gradually decreased at 3 h post-dispersion (Fig. 4c) and then gradually increased. Cellular levels of the hook FlgE were consistent during the whole periods of assays. The averages of the relative cellular amounts of each protein, quantified by densitometric analysis of the total three independent experiments, were plotted in Fig. 4e. In contrast, the Δ*degQ* cells in the first biofilms contained the apparent levels of flagellins at the beginning of dispersal (Fig. 4d). Both the degrees and patterns of FlaJ and FlgE levels in biofilms made of this mutant were the same as those made of the wild-type cells. Therefore, the observed differences in biomass of the first and second biofilms made by wild-type and Δ*degQ* strains, which were evidenced by the apparent increase in the dispersion from the first biofilms and the formation of the second biofilms by Δ*degQ* (Fig. 4a and b), were caused by the increased levels of flagellins in Δ*degQ*.

### In vitro proteolysis of FlaBs by DegQ in the absence or presence of FlaJ

**Fig. 5:**
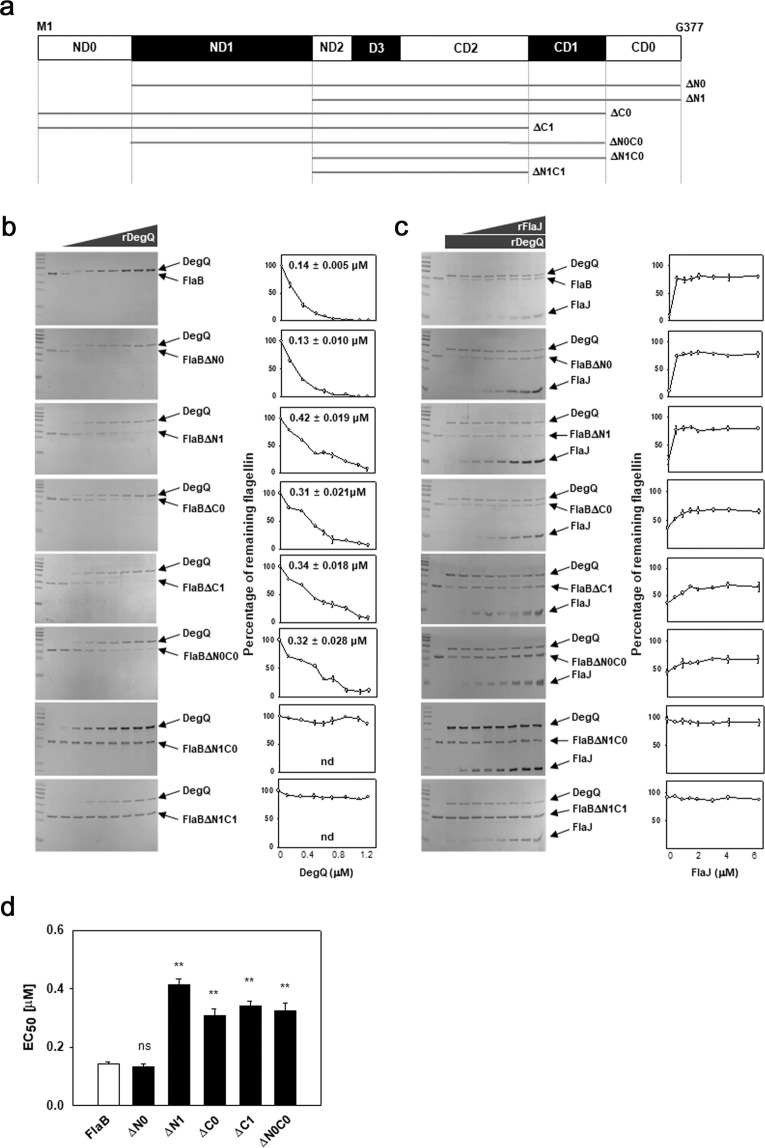
In vitro proteolysis of FlaB and its truncated polypeptides by DegQ. **a** *V. vulnificus* flagellins show to have the domains of ND0, ND1, ND2, D3, CD2, CD1, and CD0, as shown in other bacterial flagellar subunits^65^. Based upon the domain information on the FlaB (VVMO6_00808), the overexpression plasmids for seven different truncated FlaB polypeptides were constructed (Supplementary Table 1): ΔN0 (from L54 to G377; 37.5 kDa), ΔN1 (from K162 to G377; 30.6 kDa), ΔC0 (from M1 to S332; 37.8 kDa), ΔC1 (from M1 to A287; 34.5 kDa), ΔN0C0 (from L54 to S332; 35.4 kDa), ΔN1C0 (from K162 to S332; 28.5 kDa), and ΔN1C1 (from K162 to A287; 25.2 kDa). **b** Proteolysis of flagellin polypeptides by DegQ. Recombinant FlaB (1 μM) was incubated with various concentrations of rDegQ (0, 0.12, 0.3, 0.48, 0.6, 0.72, 0.9, 1.08, and 1.2 μM) as described in the “Methods”. Various derivatives of FlaB (1 μM each), of which ND0-, ND1-, CD0-, and/or CD1-domains were truncated (FlaB_ΔN0_, FlaB_ΔN1_, FlaB_ΔC0_, FlaB_ΔC1_, FlaB_ΔN0C0_, FlaB_ΔN1C0_, and FlaB_ΔN1C1_), were incubated in the same condition for proteolysis reaction. The resultant reaction mixtures were resolved in SDS-PAGE (the left gels), and the relative intensities of flagellin bands were obtained by densitometric reading, which were then plotted against the given concentrations of rDegQ with the concentration resulting in 50% proteolysis (EC_50_) (the right graphs). nd, not determined. **c** Protection of flagellin polypeptides from DegQ proteolysis by FlaJ. The proteolysis assays of rFlaB and its truncated forms were conducted in the reaction mixtures containing 1.08 μM rDegQ and various concentrations of rFlaJ (0, 0.56, 1.11, 1.67, 2.22, 3.34, 4.45, and 6.67 μM). Proteins in the reaction mixtures resolved in SDS-PAGE (the left gels) were subjected to quantitative determination using densitometry. The relative intensities of flagellin bands compared to the intensity of flagellins in the control (the second lane in each gel) were plotted against the given concentrations of rFlaJ. **d** EC_50_ values for the DegQ proteolysis. The average values of EC_50_ for proteolysis of each flagellin by rDegQ were obtained from three independent proteolysis assays. The error bars represent standard deviation. The *P-*values for comparison with the original FlaB were indicated (Student’s *t*-test; **P < 0.005; ns, not significant).

To test whether the stabilities of flagellin subunits were directly regulated by DegQ and FlaJ, in vitro proteolysis assays were carried out using recombinant proteins of a flagellin, DegQ, and FlaJ. Since FlaB and FlaD are identical in their amino acid sequences, and they constitute the major flagellin subunits of *V. vulnificus*^12^, FlaB (VVMO6_00808) was selected as a substrate for DegQ proteolysis. Reaction mixtures containing 1 μM of recombinant FlaB (rFlaB) with various concentrations of recombinant DegQ (rDegQ) ranging from 0 to 1.2 μM were analyzed by SDS-PAGE (the left gel in Fig. 5b). The intensities of the Coomassie Blue-stained bands for rFlaB, which remained after treatment of DegQ, were quantified, and the intensities of each band relative to those in the 0 μM DegQ-treated mixtures were obtained. By plotting these values against the concentrations of rDegQ, the effective concentrations of DegQ proteolyzing 50% of the added rFlaB (EC_50_) were calculated (the right graph in Fig. 5b). The degree of FlaB proteolysis by DegQ was in a DegQ dose-dependent manner, and the calculated EC_50_ for rFlaB was 0.14 μM.

The same proteolysis assays were performed in the presence of FlaJ. The reaction mixtures containing 1 μM of rFlaB and 1.08 μM of rDegQ with various concentrations of recombinant FlaJ (rFlaJ) ranging from 0 to 6.67 μM were analyzed as described above (Fig. 5c). rFlaJ in the reaction mixture was not proteolyzed by rDegQ at all. Furthermore, the cellular level of FlaJ was not affected by *degQ* mutation either (Supplementary Fig. 4). The remaining rFlaB increased in a rFlaJ concentration-dependent manner, which strongly suggested that FlaJ protected FlaB from proteolytic attack by DegQ. Indeed, almost the maximal protection by FlaJ was achieved in the reaction mixture containing 0.56 μM FlaJ, in which 77% of the added rFlaB was protected from DegQ.

To further investigate the characteristics of direct interaction between DegQ and FlaB, various truncated FlaB polypeptides were constructed by serially deleting the domains in the N-terminus (NDs) and/or C-terminus (CDs) of FlaB, as depicted in Fig. 5a. From three independent experiments of in vitro proteolysis by DegQ, the representative gels were presented in Fig. 5b, and the average values for the EC_50_ for each polypeptide were calculated and presented in Fig. 5d for comparison. When compared with the averaged EC_50_ value for rFlaB [0.13 (±0.010) μM], that for FlaBΔN0 was not changed, indicating that the ND0 domain is not involved in DegQ proteolysis. However, if both ND0 and ND1 were deleted (FlaBΔN1), its EC_50_ increased to 0.42 (±0.019) μM. Therefore, the ND1 domain was important for proteolytic interaction with DegQ. Regarding the CDs, the CD0 was important for the DegQ proteolysis, since FlaBΔC0 showed an increased EC_50_ of 0.31 (±0.021) μM, and FlaBΔC1 and FlaBΔN0C0 exhibited similar ranges of EC_50_ values of 0.34 (±0.018) μM and 0.32 (±0.028) μM, respectively. When rFlaB was truncated in the regions of both ND1 and CD0, these polypeptides, such as FlaBΔN1C0 and FlaBΔN1C1, showed almost recalcitrance to DegQ proteolysis. Moreover, all of the recombinant polypeptides derived from FlaB, except for FlaBΔN1C0 and FlaBΔN1C1, were protected if rFlaJ was provided in the reaction mixtures (Fig. 5c). The extents of protection of various truncated FlaBs by FlaJ, however, appeared to be differential, and could be divided into two groups: The first group is the mutant FlaBs truncated in ND only (FlaBΔN0 and FlaBΔN1), which showed the same pattern of protection as the original FlaB; and the second group is the CD-truncated FlaBs (FlaBΔC0, FlaBΔC1, and FlaBΔN0C0), which were less protected by FlaJ than the original FlaB. The polypeptides which belong to the latter group showed that 46–53% of the added polypeptides were protected in the presence of 0.56 μM FlaJ.

### In vitro proteolysis of flagellins and flagellin-homologous proteins

**Fig. 6:**
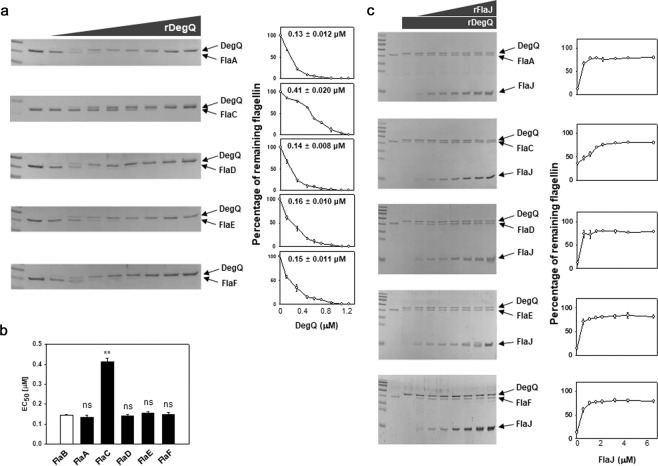
In vitro proteolysis of FlaA, FlaC, FlaD, FlaE, and FlaF. **a**, **b** Proteolysis of flagellins and flagellin-homologous proteins (FHPs) by DegQ. Recombinant proteins (1 μM each) of the flagellins (rFlaA, rFlaC, and rFlaD) and FHPs (rFlaE and rFlaF) were incubated with various concentrations of rDegQ (0, 0.12, 0.3, 0.48, 0.6, 0.72, 0.9, 1.08, and 1.2 μM). As described in Fig. 5, the remaining flagellins in reaction mixtures were resolved in SDS-PAGE (the gel images in **a**), and their relative amounts were plotted against the given concentrations of rDegQ with the calculated values of EC_50_ (the graphs in **a**). The average values of EC_50_ for proteolysis of flagellins by rDegQ were obtained from three independent assays (**b**). The error bars represent standard deviation. The *P-*values for comparison with the EC_50_ for rFlaB (derived from Fig. 5d) were indicated (Student’s *t*-test; **P < 0.005; ns, not significant). **c** Protection of flagellins and FHPs from DegQ proteolysis by FlaJ. The proteolysis assays were performed using the same recombinant proteins in the reaction mixtures containing 1.08 μM rDegQ and various concentrations (0, 0.56, 1.11, 1.67, 2.22, 3.34, 4.45, and 6.67 μM) of rFlaJ. Flagellins in the reaction mixtures resolved in SDS-PAGE (the gel images in **c**) were subjected to quantitative determination using densitometry. The relative intensities of flagellin bands were plotted against the given concentrations of rFlaJ.

In addition to FlaB, the other flagellin subunits (FlaA, FlaC, and FlaD) and the flagellin-homologous proteins (FHPs, FlaE and FlaF^12^) of *V. vulnificus* were further tested to determine if they have the same or different susceptibility to the proteolytic activity of DegQ. Recombinant proteins of FlaA (VVMO6_00809), FlaC (VVMO6_02255), FlaD (VVMO6_02252), FlaE (VVMO6_02251), and FlaF (VVMO6_00807) were prepared, and then subjected to in vitro proteolysis assays under the same conditions as described above. As shown in Fig. 6a and c, three flagellins and two FHPs were proteolyzed in a DegQ dose-dependent manner and protected in a FlaJ-concentration-dependent manner. It was not surprising that rFlaD showed the same proteolysis kinetics as FlaB, since both proteins have exactly the same amino acid sequences^12^. rFlaA and two rFHPs showed almost the same proteolysis kinetics as rFlaB or rFlaD. However, the degrees of proteolysis by DegQ and protection by FlaJ were distinct in rFlaC. The averaged EC_50_ for rFlaA, rFlaD, rFlaE, and rFlaF were 0.13 (±0.012) μM, 0.14 (±0.008) μM, 0.16 (±0.010) μM, and 0.15 (±0.011) μM, respectively (Fig. 6b). In contrast, the averaged EC_50_ for rFlaC was 0.41 (±0.020) μM, which indicated that FlaC was significantly more resistant to DegQ proteolysis than other flagellins and FHPs.

### In vitro proteolysis of the mutant FlaCs by DegQ

Since the ND1 and CD0 domains, which are involved in the proteolytic interaction with DegQ (as shown in Fig. 5), we speculated that some amino acid residues uniquely present in FlaC might be responsible for determining the differential susceptibility of FlaC to DegQ. A HtrA-typed protease has been reported to have preferred amino acids in the target substrates for its proteolytically attacking sites (i.e., Ile, Leu, Thr, Ala, Val, and Ser^24^). In silico analysis of the amino acid sequences in ND1 and CD0 revealed that FlaC was found to have distinct amino acid residues (three Met residues in ND1; a Gly and a Met in CD0; Supplementary Fig. 5a), at which other amino acids residues preferred by DegQ are positioned and conserved among FlaA, FlaB, FlaD, FlaE, and FlaF. These amino acid residues are also conserved in the FlaC of *V. parahaemolyticus* (Supplementary Fig. 5a). As a consequence, these five amino acids in FlaC were chosen to be substituted with the amino acids found in other flagellin subunits: Met65 to Val (M65V), Met157 to Val (M157V), Met159 to Leu (M159L), Gly365 to Ser (G365S), and Met380 to Leu (M380L) (Supplementary Fig. 5b). Subsequently, in vitro proteolysis assays were conducted under the same conditions described above by utilizing 1.0 μM of each recombinant polypeptide derived from FlaC.

**Fig. 7:**
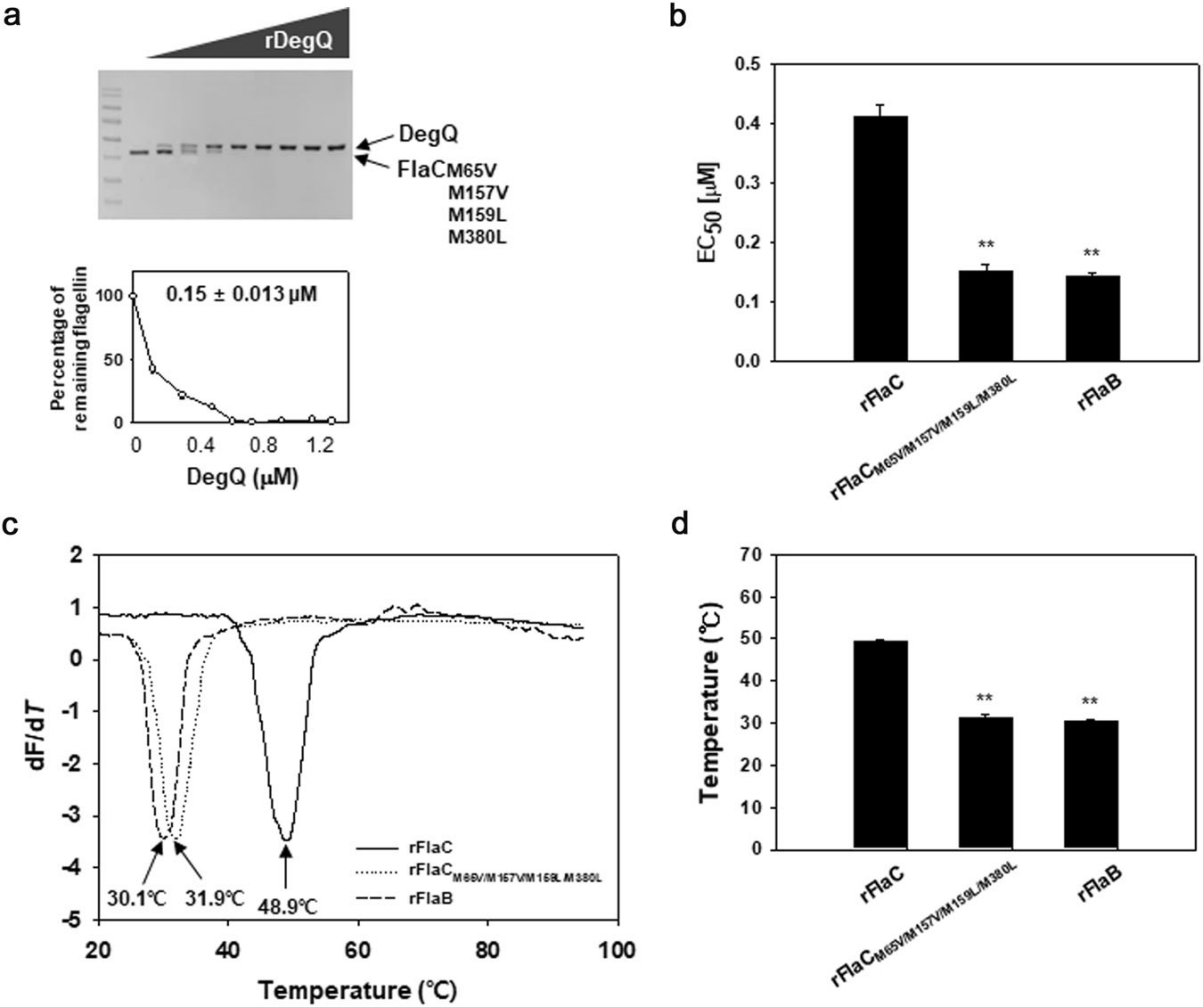
In vitro proteolysis of the mutagenized FlaC. **a**, **b** Proteolysis of FlaC_M65V/M157V/M159L/M380L_ by DegQ. Mutagenized FlaC (1 μM), in which four Met residues at the 65, 157, 159, and 380th positions were substituted with the amino acids at the corresponding positions in FlaB (as shown in Supplementary Fig. 5b), were incubated with various concentrations of rDegQ (0, 0.12, 0.3, 0.48, 0.6, 0.72, 0.9, 1.08, and 1.2 μM). As described in Fig. 5, the remaining FlaC_M65V/M157V/M159L/M380L_ in reaction mixtures were resolved in SDS-PAGE (the gel image), and their relative amounts were plotted against the given concentrations of rDegQ with the calculated values of EC_50_ (**a**). The average values of EC_50_ for proteolysis of the FlaC_M65V/M157V/M380L_ by rDegQ were obtained from three independent proteolysis assays. For comparison, those of the original rFlaC and rFlaB were included (**b**). The error bars on the bar graph represent standard deviation. The *P-*values for comparison with the original FlaC were indicated (Student’s *t*-test; **P < 0.005). **c**, **d** Determination of the stability of the original and mutagenized flagellins. Thermal denaturation profiles of rFlaB, rFlaC, and rFlaC_M65V/M157V/M159L/M380L_ were monitored by measuring the intensity of a fluorescent dye, SYPRO Orange, which had been labeled to the recombinant proteins (1 μM each), as described^63^. The melting temperatures (*T*_m_) of each recombinant polypeptide, at which the lowest value of −dF/d*T* pointed, were obtained, as described in “Methods” (**c**). The average values of *T*_m_ of each recombinant polypeptide were obtained from three independent experiments (**d**). The error bars on the bar chart represent standard deviation. The *P-*values for comparison with the original rFlaC were indicated (Student’s *t*-test; **P < 0.005).

Gly365 did not appear to be involved in the differential susceptibility to DegQ proteolysis, since FlaC_G365S_ showed the same EC_50_ [0.40 (±0.020) μM] as the original FlaC [EC_50_ of 0.41 (±0.020 μM)] (Supplementary Fig. 6a). In contrast, slightly increased susceptibility of the FlaC derivatives were observed when one of the four Met residues was mutated. FlaC_M65V_ and FlaC_M380L_ produced the EC_50_ values of 0.36 (±0.024) and 0.33 (±0.020) μM, respectively (Supplementary Figs. 6b and c). Thus, a FlaC derivative whose four Met residues were mutated to produce FlaC_M65V/M157V/M159L/M380L_, was prepared and then subjected to the same proteolysis assay (Fig. 7a). It showed highly increased susceptibility with an EC_50_ of 0.15 (±0.013) μM, which is almost the same as the EC_50_ of FlaB [0.14 (±0.005 μM)] (Fig. 7b).

To determine whether or not the four Met residues in the domains of ND1 and CD0 of FlaC actually assisted to maintain the protein stability of FlaC, melting temperatures (*T*_m_), one of the parameters determining protein stability^25^, of rFlaC were obtained from its thermal denaturation profile (Fig. 7c). This was then compared with those of rFlaB and rFlaC_M65V/M157V/M159L/M380L_. The *T*_m_ of FlaC and FlaB were estimated to be 49.2 + 0.40 °C and 30.4 + 0.41 °C, respectively (Fig. 7d). Interestingly, the *T*_m_ of rFlaC_M65V/M157V/M159L/M380L_ was 31.4 + 0.52 °C, which was close to the *T*_m_ of FlaB.

### Distinct motility characteristics determined by FlaC

**Fig. 8:**
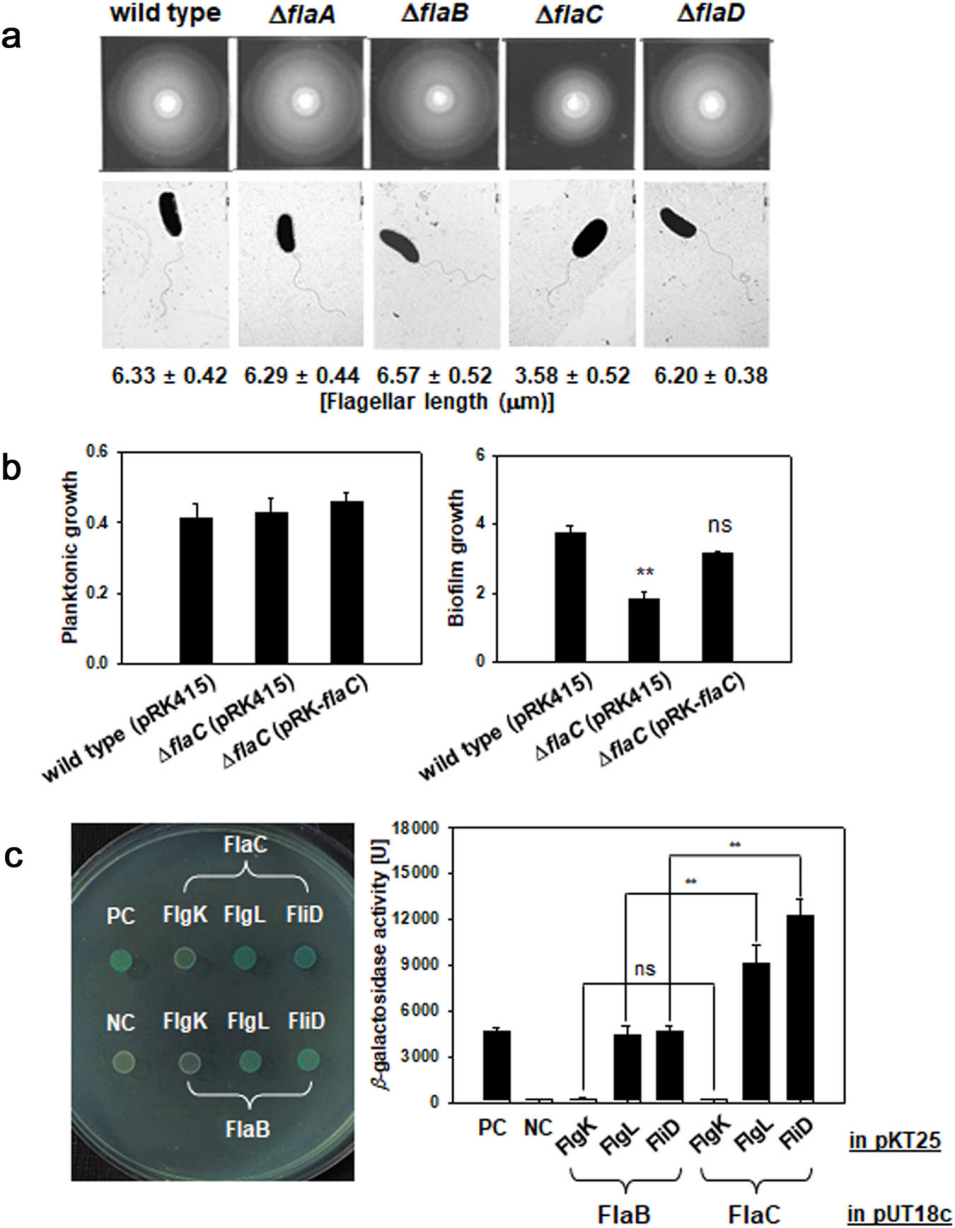
Roles of FlaC in flagellation and flagellum-mediated behaviors. **a** Swimming motility and flagellar lengths. Five microliters of the freshly grown cultures (at an OD of 1.0) of *V. vulnificus* strains of wild type, Δ*flaA*, Δ*flaB*, Δ*flaC*, and Δ*flaD* were spot-inoculated on a soft-agar plate made of LBS containing 0.3% agar and incubated at 30 °C for 6 h (upper images). Individual cells of *V. vulnificus* strains were observed under TEM to examine their polar flagella (lower images with the flagellar lengths [in μm] estimated from at least 20 cells of each strain), as described in “Methods”. **b** Biofilm formation by Δ*flaC*. Standard biofilm assay using borosilicate tubes was performed with the wild type carrying pRK415, Δ*flaC* carrying pRK415, and Δ*flaC* carrying pRK415-*flaC*. At 48 h post-inoculation, the planktonic cell density was measured by spectrophotometry at 595 nm (OD_595_), and biofilm growth was estimated by staining with crystal violet and subsequent spectrophotometry at 550 nm (OD_550_). **c** Interaction of FlaC with other components of a filament. To examine whether FlaC differentially interacts with the hook-associated proteins (FlgK and FlgL) and/or capping protein (FliD) constituting a polar flagellum^66,67^, a bacterial two-hybrid system expressing *β*-galactosidase was utilized. *E. coli* BTH101 cells containing pUT18c-*flaC*/pKT25-*flgK*, pUT18c-*flaC*/pKT25-*flgL*, or pUT18c-*flaC*/pKT25-*fliD*, were spotted on an LBS agar plate supplemented with 40 μg/ml X-gal (**a** left image). The *β*-galactosidase activities were quantified using ONPG and presented as Miller units. For comparison, pUT18c carrying the *flaB* gene instead of *flaC* was also used for combination with pKT25 carrying *flgK*, *flgL*, or *fliD*. The positive (PC) and negative controls (NC) were *E. coli* BTH101 containing pUT18c- *zip*/pKT25-*zip* and pUT18c/pKT25, respectively. The *P-*values for comparison between FlaC and FlaB were indicated (Student’s *t*-test; **P < 0.005, ns, not significant). The error bars on the bar charts represent standard deviation.

Since FlaC exhibited higher stability and more recalcitrance to proteolysis by DegQ than FlaA, FlaB, and FlaD, it was speculated that FlaC might play roles in the motility of *V. vulnificus*, which are not found in the roles of other flagellin subunits. First, to investigate the role of FlaC in motility and flagellation, both swimming ability on soft-agar plates and cellular morphology on TEM images were examined (Fig. 8a). The wild type and mutants of Δ*flaA*, Δ*flaB*, and Δ*flaD* showed the same degrees of swimming motility and had polar flagella, whose lengths ranged from 6.2 to 6.6 μm. In contrast, Δ*flaC* showed less swimming ability on soft agar and a shorter polar flagellum, with an average length of 3.6 μm. These results suggested that the flagellin subunits of FlaA, FlaB, and FlaD could be used as surrogates if one of these subunits was missed, and the mature flagellum was completely assembled to be able to swim as the wild type. However, if FlaC was missing, short flagellum was formed, and swimming motility was decreased. The reduced degree of motility of Δ*flaC* was further evidenced by measuring the growth of biofilms formed by Δ*flaC* (Fig. 8b). Its ability to form biofilm can be restored to that of the wild type if the Δ*flaC* mutant was complemented with the intact *flaC* gene in trans.

Formation of a shorter, immature flagellum by Δ*flaC* led us to hypothesize that FlaC would differentially interact with some components in the filament structure, such as HAPs. To examine this possibility, a *β*-galactosidase-based bacterial two-hybrid system was used with various combinations of a plasmid containing *flaB* or *flaC* with the other plasmid containing the gene encoding one of the HAPs, such as *flgK*, *flgL*, or *fliD* (Fig. 8c). As anticipated from the previous findings concerning the interactions of HAPs with flagellin(s) in other bacterial species^26,27^, FlaB and FlaC showed direct interactions with FlgL (HAP3) and FliD (CAP), but no interaction with FlgK (HAP1) was observed. Interestingly, FlaC showed significantly stronger interactions with both FlgL and FliD than did FlaB (the right graph in Fig. 8c). This suggested that FlaC, which is exported from the cytoplasm through a hook, might play a crucial role in initiating filament growth via strongly interacting with FlgL adjacent to the hook and FliD located at the end of a filament tip.

**Fig. 9:**
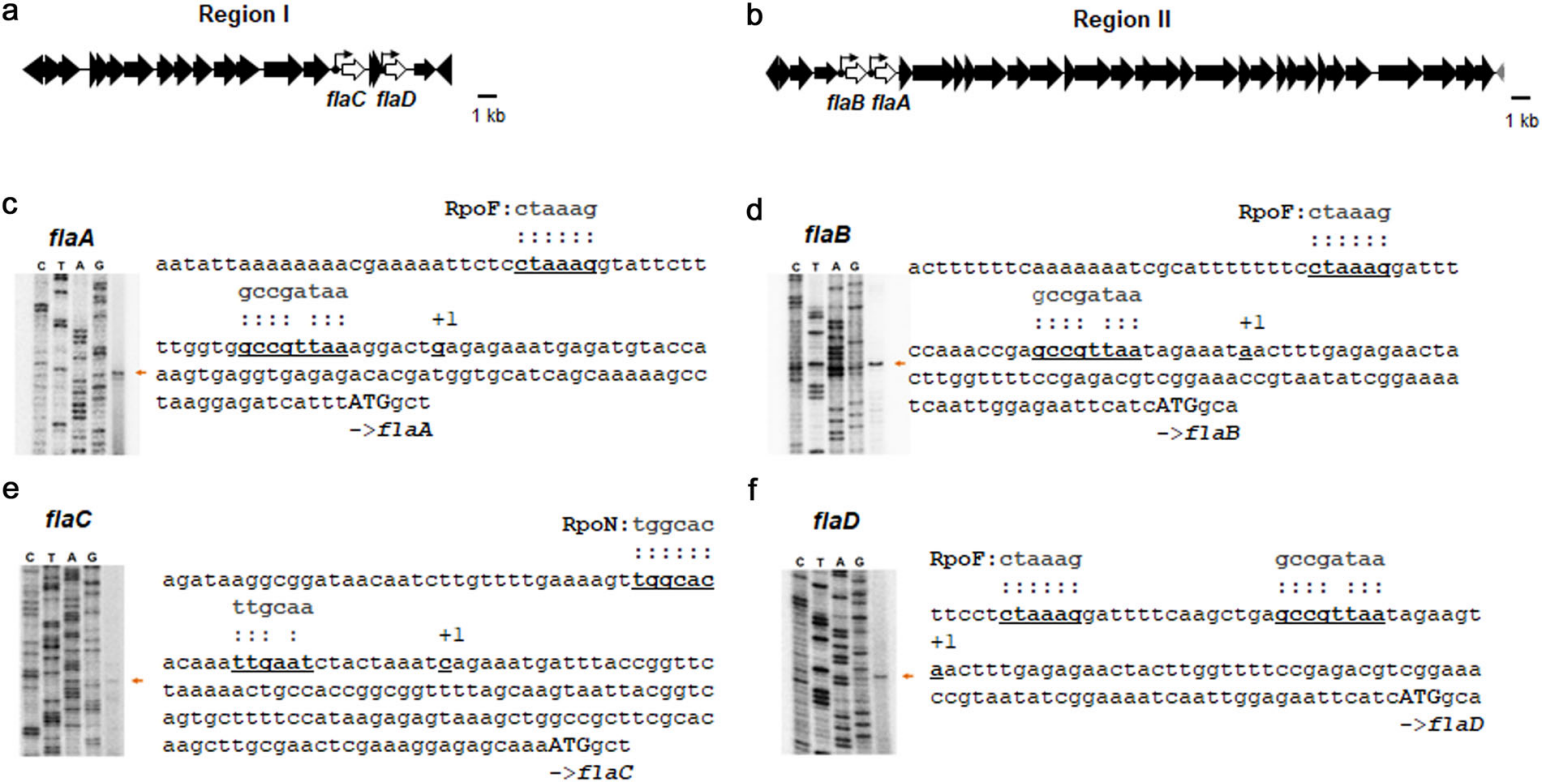
Transcription of *flaA*, *flaB*, *flaC*, and *flaD* genes. **a**, **b** Gene clusters for flagellar assembly. *V. vulnificus* MO6-24/O genomes include four flagellin genes^12^, *flaC* (VVM06_02255) and *flaD* (VVM06_02252) at region I and *flaB* (VVM06_00808) and *flaA* (VVM06_00809) at region II. **c**–**f** Identification of transcription initiation sites (TIS) and promoters. Primer-extension experiments using a primer specific to each *fla* gene (Supplementary Table 2) revealed TIS and the promoters of *flaA* (**c**), *flaB* (**d**), *flaC* (**e**), and *flaD* (**f**). Lane C, T, A, and G represent the nucleotide sequencing ladders of the upstream region for each gene. An arrow denotes the cDNA band indicating TIS. TIS (designated with +1) and its promoter (underlined and aligned with the consensus sequences for RpoN or RpoF^68,69^) were indicated in nucleotide sequences upstream of the initiation codon (ATG) of each gene.

To support this hypothesis, FlaC should be produced earlier than the other flagellin subunits. The *V. vulnificus* genes encoding the flagellin subunits locate in region I and region II, as gene arrangements of ‘*flaC-*(hypothetical ORF)_2_*-flaD*’ and ‘*flaB-flaA*’, respectively (Fig. 9a and b). A single transcription initiation site (TIS) for each gene was identified through primer-extension experiments, which indicated the promoter regions in its upstream regions. The genes of *flaA*, *flaB*, and *flaD* were shown to have promoters highly homologous to the consensus RpoF (σ^28^) promoter that includes -10 (GCCGATAA) and -35 (CTAAAG) (Fig. 9c, d, and f). In contrast, a RpoN (σ^54^)-dependent promoter, which is homologous to -12 (TTGCAA) and -24 (TGGCAC), was discernible in the upstream of the *flaC* TIS (Fig. 9e). Based upon the regulatory cascade for flagellar gene systems in *Vibrio* species^28^, RpoN locates upstream of the cascade and activates the expression of *rpoF* gene. As a consequence, it is assumed that FlaC synthesis precedes synthesis of the other flagellins in cytoplasm.

## Discussion

A serine protease, DegQ, determined the cellular levels of four flagellin subunits in *V. vulnificus* by direct proteolysis (Figs. 5b and 6a). Thus, the reduced proteolytic pressure on the nascent flagellin polypeptides is assumed to be required for *V. vulnificus* cells being flagellated when they need to be in a motile state, for example, under the conditions of the dispersal stage for biofilm formation. The cellular level of DegQ was found to be considerably decreased in cells inhabiting the biofilms being incubated under conditions inducing the dispersal stage (Fig. 4c). In addition, a flagellin-specific chaperone, FlaJ, played a role in protecting flagellins against proteolysis derived from DegQ (Figs. 5c and 6c). The cellular level of FlaJ was found to be increased under the dispersal condition (Fig. 4c). Thus, the cytoplasmic accumulation of flagellins through increased FlaJ and decreased DegQ levels, which were able to support filament growth for a complete assembly of the *V. vulnificus* polar flagellum, was acquired in the dispersing cells.

DegQ belongs to the protease family of HtrA, which was initially found as Do in *E. coli*^29^ and subsequently shown to target cytoplasmic protein, such as IciA^30^. Two additional proteases belonging to the HtrA family, besides Do (or DegP), have been further identified in *E. coli*, which include DegQ and DegS^31^. These proteases play roles in surviving *E. coli* under the conditions of elevated incubation temperatures^32^. Major targets for these proteases are denatured or mislocalized polypeptides in the periplasmic space^33,34^. In the case of *V. vulnificus*, only *degQ* and *degS* are discernible in their genomes. Amino acid sequences of *V. vulnificus* DegQ indicate that it possesses an apparent signal peptide at its N-terminus of the nascent polypeptide, suggesting that it is able to act in the periplasmic space although its substrate has not yet been reported in this bacterial species^35^. Indeed, the majority of DegQ proteins were concentrated in the periplasm, in which OmpU and PhoA were localized (Supplementary Fig. 2). However, considerable amounts of DegQ were apparently present in the cytoplasm, too. In the current study, we demonstrated that the target proteins for DegQ proteolysis are flagellin subunits. Since the flagellin subunits are speculated to be exported from the cytoplasm to the tip of the growing filament through the flagellum-specific type 3 secretion system (T3SS)^36^, flagellins are not supposed to be present in the periplasmic space. Therefore, DegQ would be able to proteolyze nascent polypeptides of four flagellin subunits in the cytoplasm. The increased levels of flagellins were accompanied by increased FlaJ (Fig. 4c and e), which counteracted the proteolytic activity of DegQ toward flagellins by specifically binding to the CD of flagellins (Fig. 5c). The fact of the cytoplasmic localization of the FlaJ chaperone further supports that the cytoplasmic fraction of DegQ would proteolyze the nascent flagellins prior to the export event through FlhA, a gate protein of the flagellum-specific T3SS^36,37^. In a previous report, the DegP purified from the cytoplasmic fraction of *E. coli* cells exhibited efficient proteolytic activity towards various proteins^38^. The recombinant DegQ used in in vitro proteolysis assays (Figs. 5–7) was a polypeptide tagged with 6-His at its leader peptide. As a consequence, in future study, it needs to be elucidated the comprehensive role of the N-terminal leaders in DegQ and its homologs prior to the translocation across the inner membranes, when they have not yet been processed in the cytoplasm.

Production of DegQ began to decrease in the beginning of the incubation condition, which could induce the dispersal of preexisting biofilms, and eventually was not detectable after 6 h (Fig. 4c and e). It might suggest the presence of a mechanism(s) sensing the specific signals or parameters representing the stages of biofilm formation. The induced expression of *degP*, under the conditions of extracytoplasmic stresses, has been shown to be controlled at the level of its transcription via RpoE, whose activities were modulated by actions of some heat-shock proteases^39–41^. In addition to RpoE, it is also regulated by a two-component regulatory system, CpxRA^42^. Although the genes of *degQ* and *degS* are mapped in an operon-like arrangement, each gene appears to be transcribed independently and their expressions are not heat-inducible in *E. coli*^41^. Signal(s) turning on or off the *degQ* gene in response to the specific stage of biofilm formation remain to be determined. Some signals have been shown to play roles in regulating the expression of genes essentially requisite for biofilm development. For example, the cellular levels of cyclic AMP and cyclic di-GMP control the transcription of the *vps* (*Vibrio* polysaccharide) operon in *V. cholerae* via specific interaction with the transcription factors, CRP and VpsR, respectively^43,44^. Cyclic di-GMP also controls the transcription of the gene clusters of the *V. cholerae* flagella via an interaction with FlrA^45^.

Stage-specific fluctuation of the cellular levels of flagellin subunits, which has been demonstrated to be regulated at the level of post-translation as mentioned above, might be additionally regulated by another regulator. As shown in Fig. 4d, under a dispersal-inducing condition, the biofilm cells constructed by Δ*degQ* showed to contain increased flagellin levels compared to the cells at the beginning of dispersal. Therefore, it is speculated that a regulator inducing the expression of flagellins might be operating in a DegQ-independent manner.

FlaC was less susceptible to proteolysis by DegQ, due to the presence of four Met residues (Met65, Met157, Met159, and Met380; Fig. 7) located in the domains of ND1 and CD0, which were required for DegQ-mediated proteolysis (Fig. 5). When these Met residues were substituted with the amino acid residues found in the corresponding positions of FlaB, the parameters indicating its proteolytic susceptibility (EC_50_ values; Fig. 7a) and thermal stability (*T*_m_; Fig. 7c) were altered to those of FlaB. Met has been reported to confer special properties in proteins due to the presence of sulfur, which can interact with nearby aromatic amino acid residues to cause a change in protein stability^46,47^. Upon oxidation of the sulfur in Met, it is converted into methionine sulfoxide that has increased strength in interaction with aromatic amino acid residues^48,49^. Moreover, a transcription factor of *E. coli*, HypT, is active in transcription of the target genes when its three Met residues are oxidized^50^. Interestingly, the degrees of reduction in EC_50_ values of the mutated FlaC polypeptides were increased, as the numbers of substituted Met in the recombinant FlaCs increased (Supplementary Fig. 6 and Fig. 7b). Therefore, it was speculated that four Met (i.e., three Met in the ND1 and a Met in the CD0 of FlaC) might augment protein stability via combinatory oxidation of four Met residues. The polar flagellum of *V. parahaemolyticus* is composed of multiple flagellin subunits^12^. Alignment of the amino acid sequences of their ND1 and CD0 with those of the *V. vulnificus* FlaC revealed that four Met are conserved in the *V. parahaemolyticus* FlaC, too (Supplementary Fig. 5a). Interestingly, transcription initiation of the *flaC* gene was reported to be dependent upon RpoN in *V. parahaemolyticus*^51^, as the *V. vulnificus flaC* did (Fig. 9c to f). In *Shewanella putrefaciens*, a flagellin subunit, FlaA, whose expression is dependent upon RpoN, was found to be more abundant in a filament close to the hook than the other RpoN-independent flagellin subunit^52^. Therefore, it is likely that the involvement of Met residues in differentially stabilizing the RpoN-dependent, early expressed flagellins might extend to the other pathogenic *Vibrio* species, which have multiple kinds of flagellin subunits for a flagellar filament.

In conclusion, the post-translational regulation of flagellins via a direct, but differential, proteolysis by DegQ determined the different cellular levels of each flagellin subunit, which in turn might be sequentially exported for the hierarchical construction of the filament of mature flagellum.

## Methods

### Bacterial strains and culture conditions

The bacteria and plasmids used in this study are listed in Supplementary Table 1. *E. coli* strains used for plasmid DNA preparation and conjugational transfer were grown in Luria-Bertani (LB) medium supplemented with appropriate antibiotics at 37 °C. *V. vulnificus* strains were grown at 30 °C in LBS (LB medium containing NaCl at a final concentration of 2.5% [w/v]) or AB medium (300 mM NaCl, 50 mM MgSO_4_, 0.2% [w/v] vitamin-free casamino acids, 10 mM potassium phosphate, 1 mM *L*-arginine, pH 7.5)^53^ supplemented with 1.0% [w/v] fumarate. Antibiotics were used at the following concentrations: for *E. coli*, ampicillin at 100 μg/ml, chloramphenicol at 20 μg/ml, kanamycin at 100 μg/ml, and tetracycline at 15 μg/ml; and for *V. vulnificus*, chloramphenicol at 4 μg/ml, kanamycin at 300 μg/ml, and tetracycline at 3 μg/ml.

### Construction of deletion mutants of *V. vulnificus*

For construction of *degQ* deletion mutant, a *degQ* upstream region of 744-bp was amplified from the genomic DNA of *V. vulnificus* MO6-24/O using two primers, degQ-upF and degQ-upR. The PCR product was then cloned into a plasmid, pBlueScript SKII(+) to produce pKdegQ01. A 1227-bp DNA fragment containing downstream region of the *degQ* gene was made using two primers, degQ-downF and degQ-downR, and cloned into the corresponding sites of pKdegQ01 to result in pKdegQ02. A 1971-bp DNA fragment of pKdegQ02 digested with SacI and SalI was ligated to a suicide vector, pDM4^54^, to generate pKdegQ03. For construction of *flaC* deletion mutant, a *flaC* upstream region of 950-bp was amplified from the genomic DNA of *V. vulnificus* MO6-24/O using two primers, flaC-upF and flaC-upR. The PCR product was then cloned into a plasmid, pBlueScript SKII(+) to produce pMflaC01. A 780-bp DNA fragment containing downstream region of the *flaC* gene was made using primers flaC-downF and flaC-downR, and cloned into the corresponding sites of pMflaC01 to result in pMflaC02. Then, 1.2-kb kanamycin resistance gene (*nptI*) was isolated from pUC4K (Pharmacia) and inserted into the BamHI site of pMflaC02 to produce pMflaC03. A 2930-bp DNA fragment of pMflaC03 digested with ApaI and SacI was ligated to a suicide vector, pDM4, to generate pMflaC04. *E. coli* SM10λ*pir* carrying pKdegQ03 or pMflaC04 was conjugated with *V. vulnificus* MO6-24/O and the exconjugants were selected on the thiosulfate citrate bile sucrose medium supplemented with 3 μg/ml chloramphenicol. Colonies with the characteristics indicating a double homologous recombination event were isolated (e.g., resistance to 5% [w/v] sucrose, sensitivity to chloramphenicol, and/or resistance to kanamycin)^55^. Deletion of the target gene in the candidate colonies was confirmed by PCR with specific primer sets and by complementation with a broad-host-range vector pRK415 harboring the intact target gene. All the plasmids and information for the primers used in this study were listed in Supplementary Tables 1 and 2, respectively.

### Preparation of various recombinant proteins and polyclonal antibodies

Various insert DNAs were prepared by amplifying the following DNA fragments: the complete ORFs for DegQ, FlaJ, and FlaA to D; several *flaB*-derived sequences truncated in various N- and/or C-terminal domains (i.e., ΔN0, ΔN1, ΔC0, ΔC1, ΔN0C0, ΔN1C0, and ΔN1C1); and several *flaC*-derived sequences having a single or multiple point mutations (i.e., M65V, G365S, M380L, and M65V/M157V/M159L/M380L). In cases of the PCR for the *flaC*-derived sequences, the overlap extension method^56^ was applied to accomplish the site-directed mutagenesis events during amplification. The lists of the primer sets for PCR are summarized Supplementary Table 2. Resultant PCR products were cloned into pQE30 and transformed into *E. coli* JM109. All the mutagenized nucleotide sequences constructed in this study were confirmed by DNA sequencing. The recombinant proteins overexpressed in the presence of 1.0 mM IPTG were purified using a nickel-nitrilotriacetic acid affinity column (Qiagen) and utilized for the in vitro proteolysis assay. Purified recombinant proteins of rFlaJ and rDegQ were used to raise the polyclonal antibodies by treating 50 μg proteins to 6-week-old female Sprague-Dawley rats. The animals received humane care in accordance with our institutional guidelines and the legal requirements (Sogang Univ. IACUCSGU2019_01).

### Western blotting analysis

Freshly grown culture of *V. vulnificus* was harvested and resuspended in PBS. Bacterial cells in resuspension were sonicated and the resultant cell lysates were separated as supernatants after centrifugation. These supernatant fractions have been examined if they contained the cellular debris including flagellar filaments, but it was not found any filament component in the fraction of cell lysates (Supplementary Fig. 7). Cell lysates prepared from wild-type and various mutant strains of *V. vulnificus* were used for sodium dodecyl sulfate-polyacrylamide gel electrophoresis (SDS-PAGE). Blotted Hybond PVDF membranes were incubated with the polyclonal antibodies (1:5000, v/v) raised against rFlaJ and rDegQ, as described above. In addition, the polyclonal antibodies specifically reacting with flagellins^12^ and FlgE^3^ were also used. Followed by treatment of the alkaline phosphatase-conjugated anti-rat IgG (1:5000, v/v), immunoreactive bands were visualized using the NBT-BCIP detection kit (Promega). The relative intensities of the protein bands on the blots from at least three independent experiments were quantified using a densitometer (Bio-Rad Gel Doc 2000 System). All blots derive from the same experiment and were processed in parallel.

### Observation of motility and flagellum

The effect of mutations on swimming motility was assessed by examining swimming motility on the LBS containing 0.3% [w/v] agar. To document swimming motility, soft-agar plates were spot-inoculated with 5 μl of cell cultures (an OD_595_ of 1.0) and incubated at 30 °C for 6 h. The diameters of the outermost rings formed by swimming wild type and mutants were compared in time-dependent manner. If necessary, a non-motile mutant, Δ*flaABCD* was inoculated on the same soft-agar plate, as a negative control. To examine the polar flagella of *V. vulnificus*, cells were observed under TEM. From the fresh cultures of various *V. vulnificus* cells grown in the AB-fumarate medium up to the mid-log phase (OD_595_ of ~1.0), bacterial cells were harvested and resuspended in 500 μl PBS (137 mM NaCl, 2.7 mM KCl, 10 mM Na_2_HPO_4_, and 2 mM KH_2_PO_4_; pH 7.4^57^). The carbon-coated, copper mesh grids, which have been pretreated with 0.1% [w/v] bovine serum albumin (BSA), were incubated with cell resuspension for 2 min. The grids were rinsed and floated on 100 μl of 1% [w/v] uranyl acetate solution^58^. The sample preparations were examined using a Philips CM100 TEM (Philips Corporation) operating at 100 kV, and the images were captured using iTEM acquisition and analysis software (Olympus Soft Imaging Solution).

### Biofilm formation assay

Overnight cultures of *V. vulnificus* were freshly inoculated to the AB-fumarate broth in borosilicate tubes. After static incubation at 30 °C for various time points, the planktonic phase in the borosilicate was removed and its cell density was measured by spectrometric reading at OD_595_. The remaining biofilms were washed with PBS and stained with 1.0% [w/v] crystal violet to estimate the degrees of biofilms formed. The stained biofilms were washed with distilled water, air-dried, and resuspended in 100% ethanol^59^. Crystal violet associated with the biofilms was quantified by spectrometric reading at OD_550_.

### Assays for bacterial adherence and dispersion

The abilities of planktonic cells to adhere on a given surface were compared by estimating the adhered cells on slide glasses. The slide glasses were submerged in 10 ml of AB-fumarate broth, which had been resuspended by either the wild type or Δ*degQ* at an OD_595_ of 0.2, for 5, 15, and 30 min without agitation. Cells attached on the slide glasses were stained with crystal violet, and their numbers were enumerated from at least 18 fields observed under a light microscope. A non-motile Δ*flaABCD* was included as a negative control. The extents of the dispersion of cells constituting the biofilms were examined by incubating the previously-formed-biofilms in excess volume of sterile PBS^60^. Biofilms formed by either wild type or Δ*degQ* around the air–liquid interface on a borosilicate tube containing 2 ml of AB-fumarate for 48 h (the first biofilms) were separated from the planktonic cells by discarding the supernatants. The remaining biofilms were washed with PBS, resuspended in 2×-volume (4 ml) of PBS, and incubated in a 30 °C-water bath for another 24 h to allow the second biofilms to be formed by the cells dispersed from the first biofilms^61^. Both the remaining first biofilms and newly formed second biofilms around the air–liquid interfaces were stained with 1.0% [w/v] crystal violet. To separately estimate two biofilms in a borosilicate tube, stained biofilms were serially eluted with 100% ethanol: After the first biofilms were resuspended in 2 ml ethanol and the associated dyes were eluted, the second biofilms were resuspended in 4 ml ethanol and the associated dyes were eluted. Eluted crystal violet was quantified by spectrometric reading at OD_550_. To monitor the changes in the cellular levels of some proteins in cells harvested from the first biofilms, the lysates were subjected to western blotting analysis of flagellins, DegQ, FlaJ, and FlgE, as described above.

### In vitro proteolysis assay

The in vitro proteolytic assay was performed by mixing various concentrations of rDegQ (0.12, 0.3, 0.48, 0.6, 0.72, 0.9, 1.08, and 1.2 μM) with 1 μM of each substrate protein (e.g., flagellin subunits or their derivatives) in 30 μl of the reaction buffer [25 mM HEPES (pH 7.5), 150 mM NaCl, 5 mM MgCl_2_, and 5 mM DTT]^62^. For the assays of protection of flagellins from DegQ proteolysis, various concentrations of rFlaJ (0.56, 1.11, 1.67, 2.22, 3.34, 4.45, and 6.67 μM) were added to the reaction buffer containing flagellins, and pre-incubated at room temperature for 1 h. Then, 1.08 μM rDegQ was added to the reaction mixtures and incubated at 37 °C for 30 min. The proteolysis reaction was stopped by adding SDS-protein loading buffer [100 mM Tris-HCl (pH 6.8), 4% SDS, 0.2% bromophenol blue, 20% glycerol, 200 mM *β*-mercaptoethanol] followed by boiling for 2 min. The resultant reaction mixtures were subjected to SDS-PAGE. To calculate EC_50_ of rDegQ for each flagellin proteolysis, quantitative determinations were obtained by densitometric reading of the corresponding flagellin bands and plotted against the given concentrations of rDegQ.

### Determination of melting temperature of recombinant proteins

Thermal stability was estimated using recombinant proteins of FlaB, FlaC, and a mutagenized FlaC (FlaC_M65V/M157V/M159L/M380L_). Each recombinant protein (1 μM) was mixed with 5× of the fluorescent dye, SYPRO Orange (Sigma-Aldrich) in a buffer consisting of 25 mM HEPES (pH 7.5), 150 mM NaCl, 5 mM MgCl_2_, and 5 mM DTT, and 10 μl of the mixtures were placed in a 96-well microplate for LightCycler 480 (Roche). While the mixtures were heated from 20 to 95 °C, the fluorescence from the dyes, which was associated with the hydrophobic regions of an unfolded protein, was measured at an excitation wavelength of 470 nm and emission wavelength of 570 nm at every 0.06 °C. Using LightCycler 480 software (Protein Melting; Roche), the values of −dF/d*T* (the first derivative of the fluorescence emission as a function of temperature) and the melting temperature (*T*_m_) of each recombinant polypeptide (pointing the lowest −dF/d*T*) were obtained^63^.

### Primer-extension analysis

Total RNA was extracted from an exponential culture (OD_595_ of 0.5) of *V. vulnificus* using the RNeasy Mini kit (Qiagen). Primer-extension reaction was performed with oligonucleotides complementary to the coding region of each gene (Supplementary Table 2). Each primer was end-labeled with [γ-^32^P] ATP using T4 polynucleotide kinase (TaKaRa) and then utilized for cDNA synthesis. RNA was converted to cDNA with the SuperScript II reverse transcriptase (Invitrogen). The resultant cDNA products were purified and resolved on a polyacrylamide gel alongside the sequencing ladders generated with the same primer used for a primer-extension reaction. The resultant gel was dried and visualized using a phosphorimager (Personal Molecular Imager FX; Bio-Rad).

### Protein-protein interaction assay using a bacterial two-hybrid system

A vector plasmid, pUT18c (Euromedex), was inserted by *flaB*^12^ or *flaC*, and the other vector plasmid, pKT25 (Euromedex), was inserted by *fliD*^12^, *flgL*^12^, or *flgK*. A set of two plasmids was cotransformed into *E. coli* BTH101. Resultant transformants were spotted on an LB agar plate containing X-gal (bromo-4-chloro-3-indolyl-β-*D*-galactopyranoside, 40 μg/ml), IPTG (0.5 mM), and appropriate antibiotics [e.g., ampicillin (100 μg/ml), kanamycin (50 μg/ml)] to observe the color development of colonies due to β-galactosidase activity. To quantify the activities of β-galactosidase, enzymatic reactions using ONPG (*O*-nitrophenol-β-galactoside, 4 mg/ml) were performed. Cells grown in LB broth containing ampicillin (100 μg/ml), kanamycin (50 μg/ml), and IPTG (0.5 mM) were resuspended in Z buffer (60 mM Na_2_HPO_4_, 40 mM NaH_2_PO_4_, 10 mM KCl, 1 mM MgSO_4_, and 50 mM mercaptoethanol), and treated with 0.1% [w/v] SDS and chloroform to prepare crude cell lysates. After adding ONPG (670 μg/ml), the reactions were stopped by the addition of Na_2_CO_3_ solution. Optical density at 420 nm was measured for each reaction mixture, and the Miller unit (U) was calculated: U = (OD_420_ × 1000)/(time × cell culture volume × OD_600_)^64^. The positive and negative controls were included in this assay, which contained pUT18c-*zip*/pKT25-*zip* and pUT18c/pKT25 (Euromedex), respectively.

### Statistical analyses

Results were expressed as means ± standard deviations of data from at least three independent experiments. Statistical analysis was performed using Student’s *t*-test (Systat Program, SigmaPlot version 9; Systat Software, Inc.). *P*-values were presented by one asterisk (*) or two asterisks (**) when 0.005 < *P* < 0.05 or *P* < 0.005, respectively.

### Reporting summary

Further information on research design is available in the Nature Research Reporting Summary linked to this article.

## Supplementary information

Supplementary Information

Reporting Summary

## Data Availability

This study includes no data deposited in external repositories. All relevant data are available from the authors.
