# Supplementary material for: Role of DegQ in differential stability of flagellin subunits in Vibrio vulnificus
Source: NPJ Biofilms Microbiomes. 2021 Apr 8;7:32. doi: 10.1038/s41522-021-00206-7 (PMC8032703; doi:10.1038/s41522-021-00206-7)
Supplement: Supplementary file 1 — Supplementary Information [file 41522_2021_206_MOESM1_ESM.pdf]

Role of DegQ in Differential Stability of Flagellin Subunits in *Vibrio vulnificus*

You-Chul Jung[1], Mi-Ae Lee[1], Han-Shin Kim[2], and Kyu-Ho Lee[1,*]

[1] Department of Life Science, Sogang University, Seoul, South Korea

[2] Department of Environmental Science, Hankuk University of Foreign Studies, Yongin, South Korea

<u>Supplementary Information</u>

I. Supplementary Figures

Supplementary Figure 1. Cellular levels of flagellins and degrees of swimming motility of various strains of *V. vulnificus*

Supplementary Figure 2. Cellular localization of DegQ proteins

Supplementary Figure 3. Swimming motilities of Δ*degQ* mutant during the early periods of incubation

Supplementary Figure 4. Effect of DegQ on FlaJ in *V. vulnificus*

Supplementary Figure 5. Methionine residues in ND1 and CD0 domains of FlaC

Supplementary Figure 6. *In-vitro* proteolysis of the mutagenized FlaCs

Supplementary Figure 7. The presence or absence of the flagellar filaments in various fractions of *V. vulnificus* culture

II. Supplementary Tables

Supplementary Table 1. Strains and plasmids used in this study

Supplementary Table 2. Oligonucleotides used in this study

III. Supplementary Reference

References

Supplementary Figure 1. Cellular levels of flagellins and degrees of swimming motility of various strains of *V. vulnificus*

(a) Ten micrograms of the cytoplasmic fractions of *V. vulnificus* lysates prepared from wild-type and various mutant strains were subjected to SDS-PAGE and subsequently western blot analysis using the polyclonal antibodies specific to *V. vulnificus* flagellin subunits, as previously described[1]. Mutant strains included Δ*degQ* (VVMO6_02600; this study), Δ*degS* (VVMO6_02601; laboratory collection), Δ*lon* (VVMO6_02121)[2], Δ*04160*; (VVMO6_04160; FtsH-like protease, laboratory collection), Δ*hslV*; (VVMO6_00298; laboratory collection), Δ*clpA*; (VVMO6_00991)[2], Δ*clpX* (VVMO6_02122)[2], and VV11275::mini-*Tn* (VVMO6_00224; YibP-like protease)[3]. Flagellins in the cytoplasm were indicated with an arrow.

(b) Five microliters of the freshly grown cultures (at an OD of 1.0) of *V. vulnificus* strains were spot-inoculated on a soft agar plate (LBS containing 0.3% agar) and incubated at 30°C for 6h.

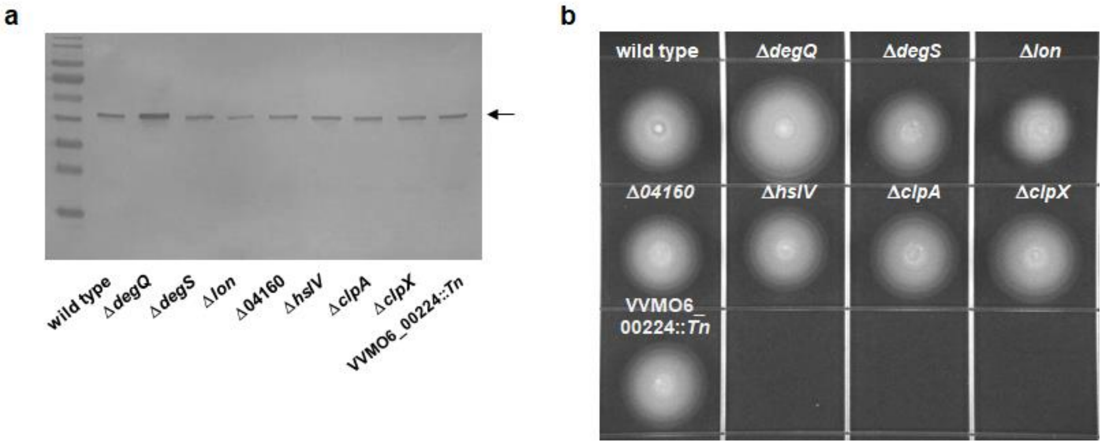

Supplementary Figure 2. Cellular localization of DegQ proteins

Cellular localization of DegQ was determined using immunofluorescence assay (IFA) and subsequent fluorescent microscopic observation. To visualize the location of membranes, an outer membrane protein, OmpU was simultaneously localized (a). For comparison, a periplasmic PhoA was localized in another set of IFA with OmpU (b). *V. vulnificus* cells attached on the poly-*L*-lysine-coated glass slides were fixed with 100% methanol at -20°C for 10 min and permeabilized with PBS/0.5% Triton X-100 for 10 min. After 1 h incubation in the PBS buffer containing 5% goat serum and 3% bovine serum albumin, the cells were further incubated overnight with anti-OmpU antibodies raised in mice (1:50, vol/vol) and anti-DegQ antibodies raised in rats (1:50, vol/vol) (a) or with anti-OmpU antibodies raised in mice (1:50, vol/vol) and anti-PhoA antibodies raised in rats (1:50, vol/vol) (b). Following three washes with PBS, the cells were incubated with Alexa Fluor 488-conjugated Goat anti-Mouse IgG (1:100, vol/vol, Molecular Probes) and Alexa Fluor 555-conjugated Goat anti-Rat IgG (1:100, vol/vol, Molecular Probes) at 37°C for 1 h, as described[4]. After the slides were mounted with VECTASHIELD Antifade Mounting Medium (Vector Laboratories), stained cells were observed under a confocal laser scanning microscope (LSM710; Carl Zeiss). Bars, 1 μm.

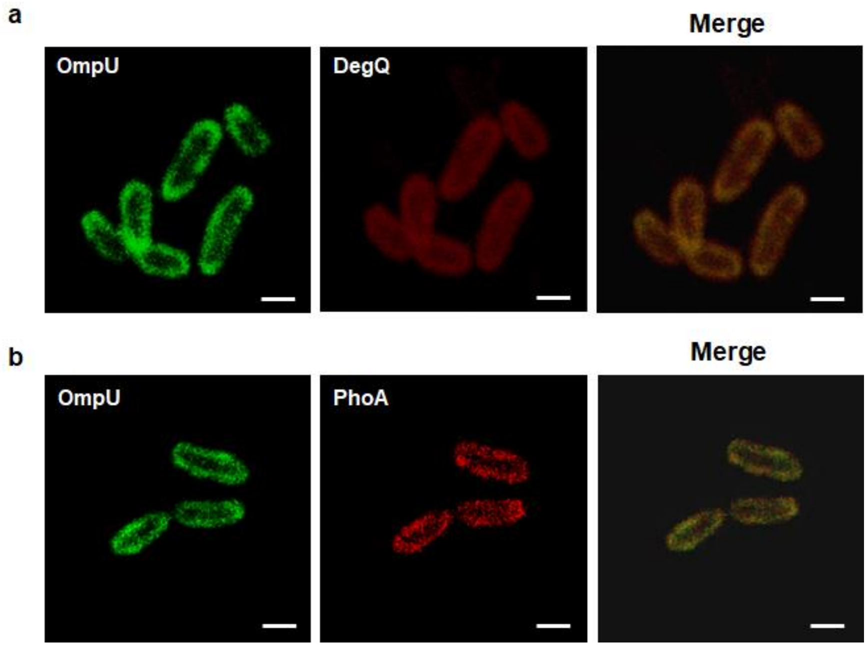

Supplementary Figure 3. Swimming motilities of Δ*degQ* mutant during the early periods of incubation

Five microliters of the freshly grown cultures (at an OD_{595} of 1.0) of *V. vulnificus* strains of wild type and Δ*degQ* were spotted on a soft agar plate (LBS containing 0.3% agar) and incubated at 30°C (a). As a negative control, a non-motile mutant, Δ*flaABCD*, was spotted on the same plate. Their motilities were compared by measuring the diameters of growing colonies at every hour during 9 h-incubation period. Measured diameters (mm) of the wild type and Δ*degQ* were presented above and those of Δ*flaABCD* were presented below the corresponding colony rings (a), and these values were plotted against incubation time (b).

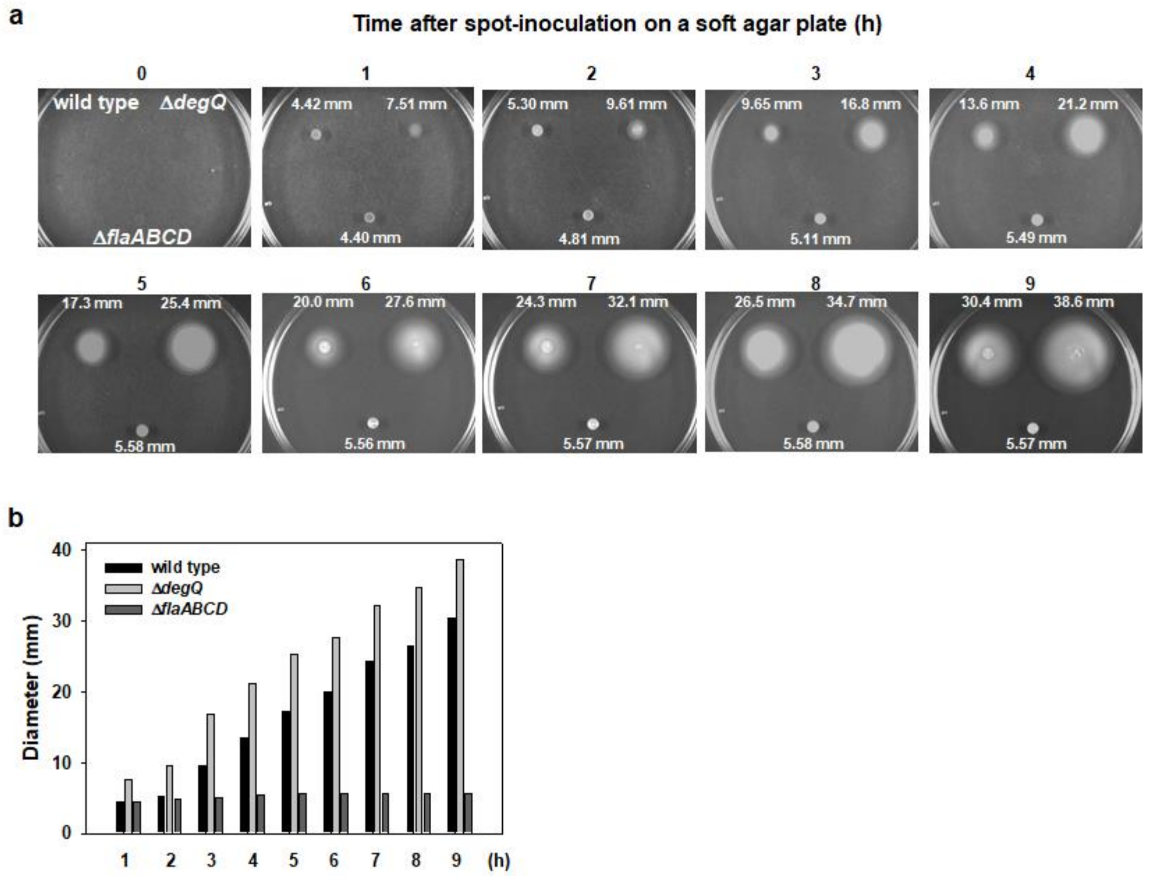

Supplementary Figure 4. Effect of DegQ on FlaJ in *V. vulnificus*

(a) Cellular levels of FlaJ in Δ*degQ* mutant. Ten micrograms of the cytoplasmic fractions of *V. vulnificus* lysates prepared from wild type, Δ*degQ*, and Δ*flaJ* were subjected to SDS-PAGE and subsequently western blot analysis using the FlaJ-specific polyclonal antibodies.

(b) *In vitro* proteolysis reaction of FlaJ in the presence of DegQ. rFlaJ (10 μM) was added to the proteolysis reaction mixture containing rDegQ (1.5 μM), as described in the Methods. The resultant reaction mixture was resolved in SDS-PAGE (lane 3). As controls, the same amounts of rDegQ (lane 1) and rFlaJ (lane 2) were loaded in the same SDS-PAGE.

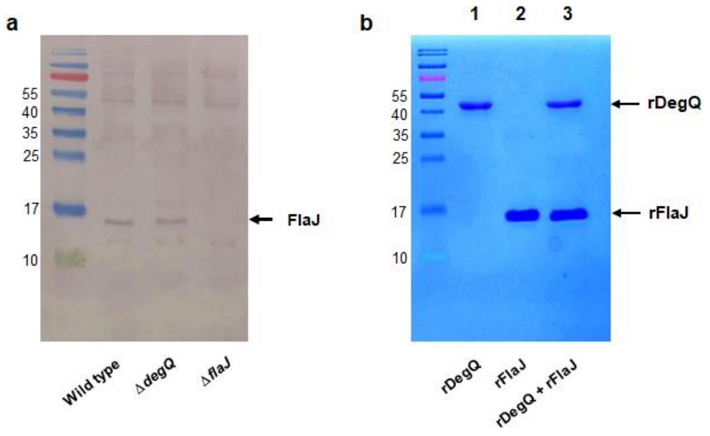

Supplementary Figure 5. Methionine residues in ND1 and CD0 domains of FlaC

(a) Amino acid sequences of the domains of ND1 and CD0 in flagellins (*e.g.*, FlaA, FlaB, FlaC, and FlaD) and flagellin-homologous proteins (*e.g.*, FlaE and FlaF) of *V. vulnificus* MO6-24/O and *V. parahaemolyticus* RIMD2210633 (GenBank accession no. of PRJNA360) were aligned. The symbols, asterisk (*), double dot (:), and single dot (.) indicate a site showing the perfect alignment, a site belonging to group exhibiting strong similarity, and a site belonging to a group exhibiting weak similarity, respectively. Four Met residues (at the 65th, 157th, 159th, and 380th positions) distinct in the FlaCs were indicated with red arrows. Another distinct amino acid residue (Gly365) was indicated with a blue arrow.

(b) Among the tentative DegQ-susceptible amino acid residues in the ND1 and CD0 of flagellins and flagellin-homologous proteins of *V. vulnificus*, the distinct five residues in FlaC were marked with the vertical lines. Overexpression plasmids for several recombinant polypeptides, of which Met65, Met157, Met159, Gly365, and Met380 have been substituted with Val, Val, Leu, Ser, and Leu, respectively, were prepared via site-directed mutagenesis using the oligonucleotide primers listed in Supplementary Table 2. These polypeptides were used for *in-vitro* proteolysis assays with rDegQ (Figure 7 and Supplementary Figure 6).

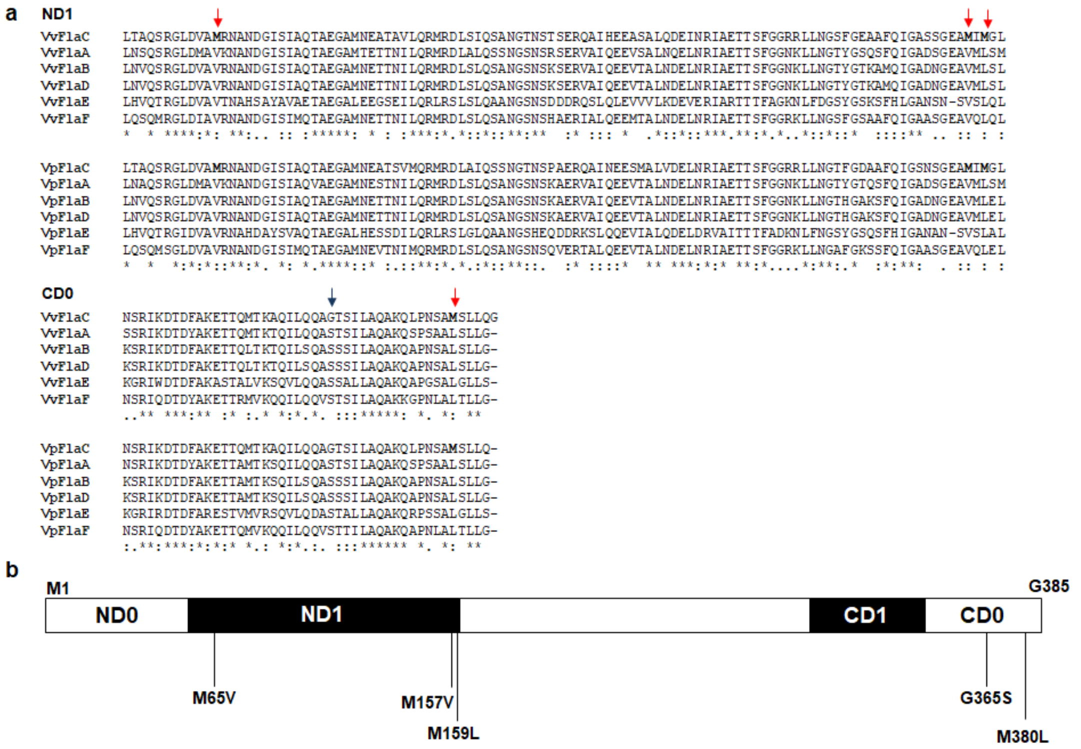

Supplementary Figure 6. *In-vitro* proteolysis of the mutagenized FlaCs

Recombinant polypeptides (1 μM each) of $FlaC_{G365S}$ (a), $FlaC_{M65V}$ (b), and $FlaC_{M380L}$ (c) were incubated with various concentrations of rDegQ (0, 0.12, 0.3, 0.48, 0.6, 0.72, 0.9, 1.08, and 1.2 μM). As described in Fig. 5, the remaining FlaCs in reaction mixtures were resolved in SDS-PAGE (the left gels), and their relative amounts were plotted against the given concentrations of rDegQ with the calculated values of EC_{50} (the right graphs).

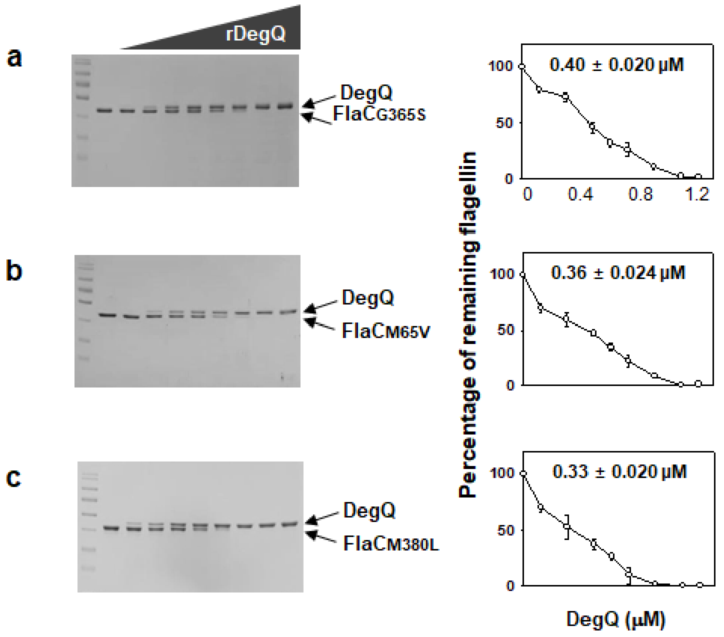

Supplementary Figure 7. The presence or absence of the flagellar filaments in various fractions of *V. vulnificus* culture

(a) A flow chart showing the procedure for preparation of a series of fractions of cell cultures. The detailed procedure was described in the Method. One hundred milliliters of freshly grown *V. vulnificus* culture (at an OD_{595} of 1.0) were centrifuged to separate the culture supernatant and a cell pellet. Protein pools in the supernatant were precipitated by treatment with 10% (vol/vol) trichloroacetic acid and resuspended in 5 ml PBS, which was designated by 'Fraction 1'. Cell pellet resuspended in 5 ml PBS was subjected to sonication to rupture the bacterial cells and subsequent centrifugation to separate the lyzed cytoplasmic fraction, which was designated by 'Fraction 2'. A pellet of debri from the rupture cells was resuspended in 10 ml PBS (Fraction 3). The half volume of the Fraction 3 was under the agitated condition in a blender for 4.5 min (at a lowest setting of a blender) to separate flagellar filaments from the membrane debri, as previously reported[1,5]. The resultant sample was then centrifuged to separate a supernatant including filaments (Fraction 4) from the pellet of debri, which was finally resuspended in 5 ml of PBS (Fraction 5).

(b) $CsCl_2$ density-gradient ultracentrifugation. Appropriate volumes of each fraction (1X, 1X, 2X, 2X, and 2X vol. of the Fractions 1, 2, 3, 4, and 5, respectively) were under the $CsCl_2$ density-gradient ultracentrifugation at 100,000 *g* and 25°C. An arrow indicates the migrating position for the flagellar filaments. These fractions were obtained using a syringe and then dialyzed against PBS for 12 h[1].

(c) Western blotting of flagellins in each fraction. Three microliters of each fraction were subjected to SDS-PAGE and the protein bands were visualized using Coomassie Brilliant blue staining (the upper panel). One microliter of each fraction separated in SDS-PAGE was subjected to western blotting analysis using the polyclonal antibodies specific to the *V. vulnificus* flagellins (the lower panel)[1]. Arrows indicate the bands of flagellin subunits.

(d) Electron microscopic observation of the Fractions 2 and 4. To examine the presence and/or absence of the flagellar filaments in the fractions, aliquots of two fractions were treated for observation under TEM, as described in Methods. Bars, 500 nm.

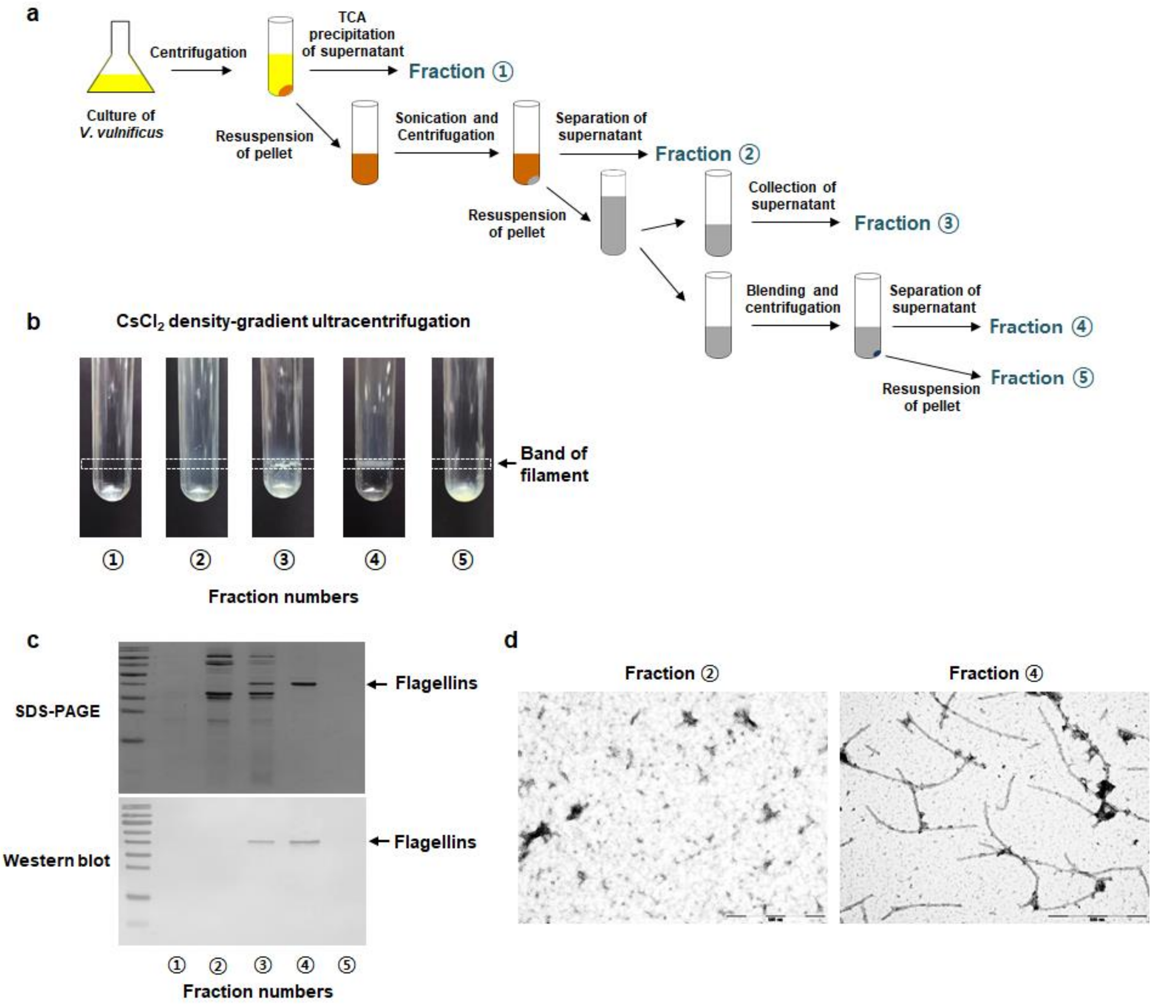
a
Culture of
V. vulnificus
Centrifugation
TCA precipitation of supernatant
Fraction ①
Resuspension of pellet
Sonication and Centrifugation
Separation of supernatant
Fraction ②
Resuspension of pellet
Collection of supernatant
Fraction ③
Blending and centrifugation
Separation of supernatant
Fraction ④
Resuspension of pellet
Fraction ⑤
b
CsCl2 density-gradient ultracentrifugation
Band of filament
① ② ③ ④ ⑤
Fraction numbers
c
SDS-PAGE
Flagellins
Western blot
Flagellins
① ② ③ ④ ⑤
Fraction numbers
d
Fraction ②
Fraction ④

Supplementary Table 1. Strains and plasmids used in this study

Strain/Plasmid	Genotype	Reference
V. vulnificus		
MO6-24/O	Clinical isolate	(6)
Δ*flaJ*	MO6-24/O, *flaJ*, Km^R	(1)
Δ*degQ*	MO6-24/O, Δ*degQ*, Km^R	This study
Δ*flaC*	MO6-24/O, Δ*flaC*, Km^R	This study
Δ*flaABCD*	MO6-24/O, Δ*flaABCD*, Km^R	(1)
E. coli		
DH5α	f80d*lacZ* DM15 *recA1 endA1 gyrA96 relA1 thi-1* *hsdR17*(r_k^- m_k^-) *supE44 deoR Δ*(*lacZYA-argF*) *U169*	Laboratory collection
SM10λpir	*thi-1 thr leu tonA lacY supE recA*:: Rp4-2-Tc::Mu*λpir*, OriT of RP4, Km^R	(7)
JM109	*endA1 recA1 gyrA96 thi-1 hsdR17*(r_k^- m_k^-) relA1 *supE44* (*lac-proAB*)[F' *traD3 6proAB lacI*^{q}*Z* M15]	Promega
BTH101	F^- *cya-99 araD139 galE15 galK16 rpsL1* (*Str*^{r}) *hsdR2* *mcrA1 mcrB1*	Euromedex
**Plasmids**		
pUC4K	pUC4 with *nptI*; Ap^R, Km^R	Pharmacia Biotech
pDM4	Suicide vector; *ori*R6K, Cm^R	(8)
pBlueScript SKII(+)	Cloning vector; Ap^R , *lac* promoter, f1, ColE1	Stratagene
pMflaC01	pBlueScript SKII(+) with 950-bp upstream region of *flaC*	This study
pMflaC02	pMflaC01 with 780-bp downstream region of *flaC*	This study
pMflaC03	pMflaC02 with 1.2-kb kanamycin resistance gene, Km^R	This study
pMflaC04	pDM4 containing XhoI and SacI fragment of pMflaC03	This study
pKdegQ01	pBlueScript SKII(+) with 744-bp upstream region of *degQ*	This study
pKdegQ02	pKdegQ01 with 1,227-bp downstream region of *degQ*	This study
pKdegQ03	pDM4 containing SacI and SalI fragment of pKdegQ02	This study
pQE30	Expression vector, Ap^R	Qiagen

pQE30-FlaA	pQE30 containing 1,131-bp *V. vulnificus flaA* ORF	(9)
pQE30-FlaB	pQE30 containing 1,134-bp *V. vulnificus flaB* ORF	(1)
pQE30-FlaC	pQE30 containing 1,158-bp *V. vulnificus flaC* ORF	(1)
pQE30-FlaE	pQE30 containing 1,128-bp *V. vulnificus flaE* ORF	(1)
pQE30-FlaF	pQE30 containing 1,134-bp *V. vulnificus flaF* ORF	(1)
pQE30-FlaD	pQE30 containing 1,134-bp *V. vulnificus flaD* ORF	This study
pQE30-DegQ	pQE30 containing 1,356-bp *V. vulnificus degQ* ORF	This study
pQE30-FlaJ	pQE30 containing 470-bp *V. vulnificus flaJ* ORF	This study
pQE30-FlaBΔN0	pQE30 containing 972-bp *V. vulnificus flaB* ORF (encoding from L54 to G377)	This study
pQE30-FlaBΔN1	pQE30 containing 648-bp *V. vulnificus flaB* ORF (encoding from K162 to G377)	This study
pQE30-FlaBΔC0	pQE30 containing 996-bp *V. vulnificus flaB* ORF (encoding from M1 to S332)	This study
pQE30-FlaBΔC1	pQE30 containing 861-bp *V. vulnificus flaB* ORF (encoding from M1 to A287)	This study
pQE30-FlaBΔN0C0	pQE30 containing 837-bp *V. vulnificus flaB* ORF (encoding from L54 to S332)	This study
pQE30-FlaBΔN1C0	pQE30 containing 513-bp V. vulnificus *flaB* ORF (encoding from K162 to S332)	This study
pQE30-FlaBΔN1C1	pQE30 containing 378-bp *V. vulnificus flaB* ORF (encoding from K162 to A287)	This study
$pFlaC_{M65V}$	pQE30 containing 1,158-bp *V. vulnificus flaC* ORF (Met65 on FlaC was substituted to Val)	This study
$pFlaC_{G365S}$	pQE30 containing 1,158-bp *V. vulnificus flaC* ORF (Gly365 on FlaC was substituted to Ser)	This study
$pFlaC_{M380L}$	pQE30 containing 1,158-bp *V. vulnificus flaC* ORF (Met380 on FlaC was substituted to Leu)	This study
$pFlaC_{M65V/M157V/M159L/M380L}$	pQE30 containing 1,158-bp *V. vulnificus flaC* ORF (Met65, Met157, Met159 and Met380 on FlaC were substituted to Val, Val, Leu and Leu, respectively)	This study

pRK415	IncP *ori*, broad-host-range vector *oriT* of RP4, Tc^R	(10)
pRK415-flaC	pRK415 with 1,167-bp VVMO6_02255	This study
pRK415-degQ	pRK415 with 1,367-bp VVMO6_02600	This study
pRK415-flaJ	pRK415 with 411-bp VVMO6_00813	This study
pKT25	BACTH plasmid for T25 fragment of adenylate cyclase (Km^r)	EUROMEDEX
pUT18c	BACTH plasmid for T18 fragment of adenylate cyclase (Ap^r)	EUROMEDEX
pKT25-zip	BACTH plasmid for T25 fragment fused to GCN4 leucine zipper	EUROMEDEX
pUT18c-zip	BACTH plasmid for T18 fragment fused to GCN4 leucine zipper	EUROMEDEX
pKT25-FlgK	pKT25, T25-*flgK* fusion	This study
pKT25-FlgL	pKT25, T25-*flgL* fusion	(1)
pKT25-FliD	pKT25, T25-*fliD* fusion	(1)
pUT18c-FlaB	pUT18c, T18-*flaB* fusion	(1)
pUT18c-FlaC	pUT18c, T18-*flaC* fusion	This study

Supplementary Table 2. Oligonucleotides used in this study

Strain/Gene	Primer name	Sequence (5'->3'; Restriction sites underlined)
**For construction of deletion mutants**		
Δ*flaC*	flaC-upF	CCCTCGAGCTTGAATCGCCACGCGCTCAGA
	flaC-upR	CGGGATCCTCTGCAATGTCACTATTGCAGGGC
	flaC-downF	CGGGATCCCGTTAGTGCTTACTGTTACAGCC
	flaC-downR	CGAGCTCTCGGTGTCTGATGCCAATGCC
Δ*degQ*	degQ-upF	CGAGCTCGTTGTCGTATCACGACTTACCACGCC
	degQ-upR	GCTCTAGAGCGTTCTACCTGTTTACCTTCAACCG
	degQ-downF	GCTCTAGAATCATTATCGGTGTCAACCGTCAGCG
	degQ-downR	ACGCGTCGACCAATATCCATCACACTCTGACGACCTTGG
**For construction of complementation plasmids**		
flaC	flaC-comF	CGGGATCCATGGCTGTAACAGTAAGCACTAACG
	flaC-comR	GGAATTCGGATTACTATTAGCCCTGCAATAGTG
flaJ	flaJ-comF	CGGGATCCAGAAGTTTACGTAAAAGACG
	flaJ-comR	GCTCTAGACCCTGACCAGATCTTTGACC
degQ	degQ-comF	GCCAAGCTTATGAAAAAACCTTTGCTTGTTTTG
	degQ-comR	GATCCTGCAGTTGAGGCGATATGTTTACTTAGCG
**For construction of overexpression plasmids for recombinant proteins**		
FlaD	flaDexp-F	CGGGATCCATGGCAGTGAATGTAAATACAAACGTAGCAGC
	flaDexp-R	AACTGCAGCCGAAATTAACCGCTGCTGCTCAGATATTAGCC
DegQ	degQexp-F	CGGGATCCATGAAAAAACCTTTGCTTGTTTTGACTGCATTG
	degQexp-R	GATCAAGCTTTTAGCGAATGACTAAATAGATAGTGC
FlaJ	flaJexp_F	CGGGATCCATGCGCGGTTCATTACAGGC
	flaJexp_R	CCCAAGCTTCCAAGCAATTAGGAGGTGTG
FlaBΔN0	flaBΔN0-F	CGGGATCCATGTTGAACGTACAAAGTCGCGGTCTAGAC
	flaB-R	AACTGCAGTCTGTCTAGTTAAGGCGATTAGCC
FlaBΔN1	flaBΔN1-F	CGGGATCCATGTCCGCGAACGGCTCAAACCTAAAATC
	flaB-R	AACTGCAGTCTGTCTAGTTAAGGCGATTAGCC

FlaBΔC0	flaB-F	CGGGATCCATGGCAGTGAATGTAAATACAAACG
	flaBΔC0-R	AACTGCAGAATCGACGCGTTCACGTTTTCGTTGATG
FlaBΔC1	flaB-F	CGGGATCCATGGCAGTGAATGTAAATACAAACG
	flaBΔC1-R	AACTGCAGAATCGCACCTTGTACGGTTGTCACGTC
FlaBΔN0C0	flaBΔN0-F	CGGGATCCATGTTGAACGTACAAAGTCGCGGTCTAGAC
	flaBΔC0-R	AACTGCAGAATCGACGCGTTCACGTTTTCGTTGATG
FlaBΔN1C0	flaBΔN1-F	CGGGATCCATGTCCGCGAACGGCTCAAACCTAAAATC
	flaBΔC0-R	AACTGCAGAATCGACGCGTTCACGTTTTCGTTGATG
FlaBΔN1C1	flaBΔN1-F	CGGGATCCATGTCCGCGAACGGCTCAAACCTAAAATC
	flaBΔC1-R	AACTGCAGAATCGCACCTTGTACGGTTGTCACGTC
FlaC$_{M65V}$	flaC-F	CGGGATCCATGGCTGTAACAGTAAGCACTAACG
	flaC$_{M65V}$-F	GGCTTAGATGTGGCGGTGCGTAATGCC
	flaC$_{M65V}$-R	GGCATTACGCACCGCCACATCTAAGCC
	flaC-R	CCCAAGCTTTTAGCCCTGCAATAGTGACATTGCAGA
FlaC$_{G365S}$	flaC-F	CGGGATCCATGGCTGTAACAGTAAGCACTAACG
	flaC$_{G365S}$-F	GCACAGATTTTGCAACAGGCAGGTACTTCG
	flaC$_{G365S}$-R1	CGAAGTACCTGCCTGTTGCAAAATCTGTGC
	flaC $_{G365S}$-R2	GGAATCGTGGAAGGTGCACATCGG
	flaC-R	CCCAAGCTTTTAGCCCTGCAATAGTGACATTGCAGA
FlaC$_{M380L}$	flaC-F	CGGGATCCATGGCTGTAACAGTAAGCACTAACG
	flaC$_{M380L}$-R	CCCAAGCTTTTAGCCCTGCAATAGTGACAGTGCAGA
FlaC$_{M65V/M157V/M159L/M380L}$	flaC-F	CGGGATCCATGGCTGTAACAGTAAGCACTAACG
	flaC$_{M65V}$-F	GGCTTAGATGTGGCGGTGCGTAATGCC
	flaC$_{M65V}$-R	GGCATTACGCACCGCCACATCTAAGCC
	flaC$_{M157VM159L}$-F	TCTGGTCAAGCGGTGATTCTGGGCTTA
	flaC$_{M157VM159L}$-R	TAAGCCCAGAATCACCGCTTCACCAGA
	flaC$_{M380L}$-R	CCCAAGCTTTTAGCCCTGCAATAGTGACAGTGCAGA
**For construction of Primer extension experiments**		
flaA	flaA_p-R	GCTCTAGAGCGGTCATTGCTGACACGTTAGTG
flaB	flaB_p-R	GCTCTAGAGGTAACGCTGTGCTGTCATTGC
flaC	flaC_p-R	GCTCTAGAGCGGATACGTTAGTGCTTACTG

flaDE	flaDE_p-R	GCTCTAGACTGTGCTGTCATTGCTGCTACG
**For construction of Bacterial Two-Hybrid system plasmids**		
flgK	pKT25-FlgK-F	AACTGCAGGGATGGCGTCGGATCTTCTGAATGTAGG
	pKT25-FlgK-R	CGGGATCCCTATCTTAATTGCAAAATGGTG
flaC	pUT18c-FlaC-F	AACTGCAGAAGCTTGCGAACTCGAAAGGAGAGC
	pUT18c-FlaC-R	CGGGATCCCACAGGATTACTATTAGCCCTGC

References

1. Jung, Y. C., Lee, M. A. & Lee, K. H. Role of flagellin-homologous proteins in biofilm formation by pathogenic *Vibrio* species. *mBio* **10**, e01793-19 (2019).

2. Lee, K. J., Jung, Y. C., Park, S. J. & Lee, K. H. Role of heat shock proteases in quorum-sensing-mediated regulation of biofilm formation by *Vibrio* species. *mBio* **9**, e02086-17 (2018).

3. Kim, H. S., Lee, M. A., Chun, S. J., Park, S. J. & Lee, K. H. Role of NtrC in biofilm formation via controlling expression of the gene encoding an ADP-glycero-manno-heptose-6-epimerase in the pathogenic bacterium, *Vibrio vulnificus*. *Mol Microbiol* **63**, 559-574 (2007).

4. Kim, J., Lee, H. Y., Lee, K. H. & Park, S. J. Phosphorylation of serine 148 in *Giardia lamblia* end-binding 1 protein is important for cell division. *J Eukaryot Microbiol* **64**, 464-480 (2017)

5. Yoon, S. S. & Mekalanos, J. J. Decreased potency of the *Vibrio cholerae* sheathed flagellum to trigger host innate immunity. *Infect Immun* **76**, 1282-1288 (2008).

6. Wright, A. C., Simpson, L. M., Oliver, J. D. & Morris, J. G. Phenotypic evaluation of acapsular transposon mutants of *Vibrio vulnificus*. *Infect Immun* **58**, 1769-1773 (1990).

7. Simon, R., Priefer, U. & Puhler, A. A broad host range mobilization system for *in vivo* genetic engineering: transposon mutagenesis in gram negative bacteria. *Nat Biotechnol* **1**, 784-791 (1983).

8. Milton, D. L., O'Toole, R., HÖrstedt, P. & Wolf-Watz, H. Flagellin A is essential for the virulence of *Vibrio anguillarum*. *J Bacteriol* **178**, 1310-1319 (1996).

9. Lee, H. J., Kim, J. A., Lee, M. A., Park, S. J. & Lee, K. H. Regulation of haemolysin (VvhA) production by ferric uptake regulator (Fur) in *Vibrio vulnificus*: repression of *vvhA* transcription by Fur and proteolysis of VvhA by Fur-repressive exoprotease. *Mol Microbiol* **88**, 813-826 (2013).

10. Keen, N. T., Tamaki, S., Kobayashi, D. & Trollinger, D. Improved broad-host-range plasmids for DNA cloning in Gram-negative bacteria. *Gene* **70**, 191-197 (1988).
